# Lipidomic-Based Algorithms Can Enhance Prediction of Obstructive Coronary Artery Disease

**DOI:** 10.1021/acs.jproteome.4c00249

**Published:** 2024-07-15

**Authors:** Thomai Mouskeftara, Olga Deda, Theodoros Liapikos, Eleftherios Panteris, Efstratios Karagiannidis, Andreas S. Papazoglou, Helen Gika

**Affiliations:** † Laboratory of Forensic Medicine and Toxicology, School of Medicine, Aristotle University of Thessaloniki, 54124 Thessaloniki, Greece; ‡ Biomic_AUTh, CIRI-AUTH Center for Interdisciplinary Research and Innovation Aristotle University of Thessaloniki, 57001 Thessaloniki, Greece; § Second Department of Cardiology, General Hospital “Hippokration”, Aristotle University of Thessaloniki, Konstantinoupoleos 49, 54642 Thessaloniki, Greece; ∥ Athens Naval Hospital, 11521 Athens, Greece

**Keywords:** lipidomics, cardiovascular disease, SYNTAX score, machine learning, XGBoost

## Abstract

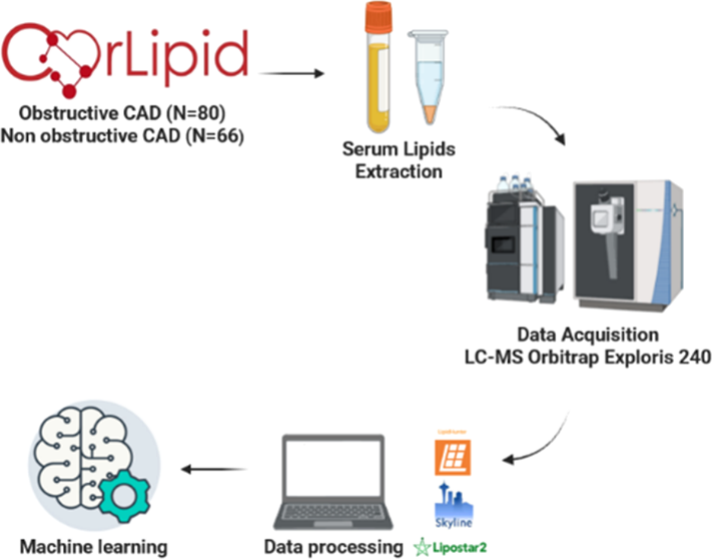

Lipidomics emerges as a promising research field with the potential to help in personalized risk stratification and improve our understanding on the functional role of individual lipid species in the metabolic perturbations occurring in coronary artery disease (CAD). This study aimed to utilize a machine learning approach to provide a lipid panel able to identify patients with obstructive CAD. In this posthoc analysis of the prospective CorLipid trial, we investigated the lipid profiles of 146 patients with suspected CAD, divided into two categories based on the existence of obstructive CAD. In total, 517 lipid species were identified, from which 288 lipid species were finally quantified, including glycerophospholipids, glycerolipids, and sphingolipids. Univariate and multivariate statistical analyses have shown significant discrimination between the serum lipidomes of patients with obstructive CAD. Finally, the XGBoost algorithm identified a panel of 17 serum biomarkers (5 sphingolipids, 7 glycerophospholipids, a triacylglycerol, galectin-3, glucose, LDL, and LDH) as totally sensitive (100% sensitivity, 62.1% specificity, 100% negative predictive value) for the prediction of obstructive CAD. Our findings shed light on dysregulated lipid metabolism’s role in CAD, validating existing evidence and suggesting promise for novel therapies and improved risk stratification.

## 1 Introduction

Coronary artery disease (CAD) constitutes the leading cause of mortality worldwide.^1,2^ Alterations in plasma lipoproteins and lipids including high-density lipoprotein cholesterol (HDL-c), low-density lipoprotein cholesterol (LDL-c), and triglycerides (TG) have been established as the primary lipid targets in the management of patients with CAD and have been traditionally utilized as risk stratifiers.^3^ Nevertheless, a thorough investigation of the circulating lipids could improve CAD risk stratification and the understanding of the underpinning mechanisms,^4^ as the accumulation of lipids in blood vessel walls drives the pathogenesis of CAD.^5^

Untargeted lipidomic analysis holds promise to further improve CAD risk prediction and may also provide prognostic information not captured by the established CAD risk factors.^6^

In the Bruneck study, plasma samples from 685 patients with CAD were analyzed with shotgun lipidomics. One hundred thirty-five lipid species were identified, providing molecular lipid signatures for cardiovascular disease and representing a promising set of new biomarkers that surpass the conventional biochemical measurements of lipid classes currently employed in clinical settings.^7^ Several studies, both smaller case-control cohorts and large-scale prospective studies, have revealed that many lipid species may be involved in the development and progression of CAD, such as acylcarnitines,^8^ fatty acids,^9^ phospholipids,^10^ and glycerolipids.^11^ Specifically, lipid species derived from the sphingolipid class, such as certain ceramide molecules, have emerged as novel risk factors in the prediction and prognosis of CAD.^12−14^ The altered sphingolipid synthesis and metabolism in CAD are caused by biochemical processes still incompletely understood. In general, increased cardiac remodeling and dysfunction have been linked to higher ceramide concentrations in plasma.^15^

Lipidomic analyses provide high-dimensional data sets; thus, the nonlinear relationships and between-variable interactions limit the applicability of traditional regression modeling for lipidomic-based risk prediction. Recent studies used more sophisticated machine learning (ML) approaches to address this issue. ML methods such as extreme gradient boosting (XGBoost), selection operator (LASSO) for traditional regression models, and artificial neural networks can improve the statistical handling of lipidomic data sets including numerous parameters.^16^ More specifically, Alshehry et al. applied weighted cox regression to correlate 310 lipid species with sudden cardiac death,^17^ while Ottosson et al. used a combination of the LASSO algorithm for variable selection and cox regression to develop a prediction model, aiming to investigate 184 lipid species and their relationship with coronary revascularization, myocardial infarction, and sudden cardiac death.^18^ Nevertheless, to the authors’ knowledge, the XGBoost approach has been scarcely used in previous lipidomic studies predictive of cardiovascular disease.^19−22^

In this study, the comprehensive information acquired by the serum lipidomic signatures was explored in relation with their predictive ability for obstructive CAD detection. The lipid profile in a subgroup of CorLipid participants was investigated, including 146 patients with suspected CAD, categorized based on their SYNTAX score (SS) values into those with and without obstructive CAD. The aim was to evaluate the utility of 288 quantified serum lipid species for the development of a ML predictive algorithm able to determine the severity of CAD. The objectives were to both characterize the normal circulating lipid metabolome and determine the changes that occurred in patients with CAD.

## 2 Methods

### 2.1 Study Population

In this study, a subgroup of 146 patients enrolled in the investigator-initiated, prospective, noninterventional ≪CorLipid≫ cohort (ClinicalTrials.gov; identifier: NCT04580173) was studied. Every trial process was carried out in accordance with the International Conference on Harmonization Guidelines for Good Clinical Practice and the Helsinki Declaration’s guiding principles.^23^ The study was approved by the local ethics committee, the Scientific Committee of AHEPA University Hospital with the reference number 12/13–06–2019, and every patient attested a written informed consent before being enrolled in the study. The participants comprised individuals with suspected CAD undergoing invasive coronary angiography by experienced interventionalists. The SS was determined in these patients, leading to their classification into two groups: individuals with obstructive CAD (SS > 0, *N* = 80) and those without obstructive CAD (SS = 0, *N* = 66). Serum samples were collected prior to coronary angiography execution and were analyzed to obtain their lipidomic profile. The samples were stored at −80 °C upon their collection.

### 2.2 Chemicals and Materials

Methanol (MeOH), acetonitrile (MeCN), isopropanol (i-PrOH), deionized water (ddH_2_O), and formic acid (all ULC/MS-CC/SFC grade) were obtained from Biosolve (Valkenswaard, Netherlands). Methyl-*tert*-butyl-ether (MTBE; ≥99%) and 3,5-di-*tert*-4-butylhydroxytoluene (BHT) were purchased from Sigma-Aldrich (Taufkirchen, Germany). Ammonium formate (NH_4_HCO_2_; MS grade) was purchased from Fluka Analytical (München, Germany). SPLASH LIPIDOMIX was purchased from Avanti Polar Lipids (Avanti Polar Lipids, Inc., Alabama).

### 2.3 Sample Preparation and Analysis

For the lipidomic analysis, 50 μL of serum samples were thawed on ice for 1 h. Five μL of SPLASH LIPIDOMIX was added to each sample. The samples were vortexed and incubated on ice for 15 min. For lipid extraction, 375 μL of ice-cold MeOH and 1250 μL of ice-cold MTBE were added, following vortexing. Samples were incubated for 1 h at 4 °C (orbital shaker, 0.20 g). Phase separation was induced by adding 375 μL of ice-cold H_2_O and the samples were shaken for 10 min at 4 °C (orbital shaker, 0.20 g). All solvents contained 0.01% (w/v) BHT and the extraction was performed on ice. After the end of incubation, samples were centrifuged for 10 min at 4 °C and 11.180 g. The organic phase was collected, transferred to 2 mL Eppendorf tubes, and evaporated to dryness *in vacuo* (Matrin Christ vacuum concentrator Christ_RVC 2–25 PLUS, 30 mbar). The individual samples were reconstituted to a final volume of 400 μL of i-PrOH. A quality control (QC) sample was prepared by mixing equal volumes of each serum sample. Diluted QCs (1:5, 1:10, 1:25, 1:50, 1:75, 1:100) in i-PrOH were also prepared and analyzed to evaluate the dynamic range of the MS response of quantified lipids. Group-specific QCs were prepared by combining equal volumes of serum samples from the same group to enhance the identification procedure.

Due to the substantial number of samples, the process of sample preparation spanned across a period of 5 days. Throughout this duration, an average of 30 samples and two batch QC samples were extracted and prepared per day. The samples were analyzed in a randomized order, with QC samples being analyzed every 10 individual samples, resulting in a total of 17 QC samples being analyzed. Initially, blank samples, ten QCs for equilibration, and diluted QCs were analyzed. In addition, group-specific QCs were also injected in positive and negative modes, followed by the analysis of individual samples only in positive mode.

### 2.4 Liquid Chromatography–Mass Spectrometry

An UHPLC-Orbitrap MS system was used for the lipidomic analysis. Separation was performed on a Vanquish Horizon system (Thermo Fisher Scientific, Germering, Germany) equipped with an Accucore C30 column (150 × 2.1 mm^2^: 2.6 μm, 150 Å, Thermo Fisher Scientific, Sunnyvale, CA) at 50 °C. The mobile phase system consisted of solvents A (MeCN/H_2_O, 1:1, v/v) and B (i-PrOH/MeCN/H_2_O, 85:10:5, v/v/v), both containing 5 mM HCOONH_4_ and 0.1% (v/v) formic acid. Elution was performed at a flow rate of 0.3 mL/min using the following elution program: 0–20 min −10 to 86% B, 20–22 min −86 to 95% B, 22–26 min −95% B isocratic, 26–26.1 min −95 to 10% B, 26.1–34.0 min −10% B isocratic, column re-equilibration. The autosampler temperature was set at 10 °C.

The UHPLC system was coupled to an Orbitrap Exploris 240 mass spectrometer (Thermo Fisher Scientific, Germering, Germany), equipped with a HESI probe. The MS data for QCs and group-specific QCs were acquired in positive and negative ionization modes using the following settings in ESI: sheath gas: 40 arb. units, auxiliary gas: 10 arb. units, sweep gas: 1 arb. unit, spray voltage: 3.5 kV (positive ion mode) and −2.5 kV (negative ion mode), ion transfer tube temperature: 300 °C, S-lens RF level: 35%, and aux gas heater temperature: 370 °C. Data were acquired in data-dependent acquisition (DDA) mode with a survey scan resolution of 120.000 (at *m*/*z* 200), an AGC target of 1 × 10^6^, a maximum IT of 100 ms in a scan range of *m*/*z* 200–1.200. Data-dependent MS2 was acquired with a resolution setting of 15.000 at 200 *m*/*z*, an AGC target of 1 × 10^5^ counts, a maximum IT of 60 ms, isolation window 1.2 *m*/*z*, and stepped normalized collision energies of 17, 27, and 37%. Dynamic exclusion for 6s, all isotopes and charge states >1 were excluded. For quantification, serum lipid extracts were analyzed in full MS mode in the positive ion mode at the resolution of 120.000 at *m*/*z* 200, an AGC target of 1 × 10^6^, and a maximum IT of 100 ms in the mass range from *m*/*z* 200–1.200. All data were acquired in profile mode.

### 2.5 Lipid Identification

Lipid annotation was performed using two software tools, Lipostar2 (version 2.0.2 Molecular Discovery Ltd., Hertfordshire, U.K.) equipped with the LIPID MAPS structure database (version September 2021)^24^ and LipidHunter 2.^25^ The raw files were imported to Lipostar directly and aligned using default settings (Thermo DDA pos and neg, respectively). Automatic peak picking was performed with the Savitzky–Golay algorithm, using the following parameters: window size set to 7, degree to 2, multipass iterations to 1, and the minimum S/N ratio was 3. Mass tolerance settings were set to 10 ppm with an RT tolerance of 0.2 min. Filters Retain lipids with an isotopic pattern and Retain lipids with MS/MS were applied. Following parameters were used for lipid identification: 5 ppm precursor ion mass tolerance and 20 ppm product ion mass tolerance. The automatic approval was performed to keep structures with a quality of 3–4 stars. All proposed identifications were manually evaluated and further filtered out to achieve the highest confidence in identification.

Raw files were converted into the mzML format using MSConvert from the Proteowizard project (version 3.0.9134). LipidHunter 2 was used to identify phospholipids in negative mode ([M + HCOO]^−^ for PC and [M – H]^−^ for all others) and sphingolipids and glycerolipids ([M + H]^+^ and [M + NH_4_]^+^, respectively) in positive mode.^25^ Lipids were identified using the following parameters: mass accuracy at the MS level −5 ppm, mass accuracy at the MS/MS level −20 ppm, precursor window ±0.75 *m*/*z*, ms level threshold 1.000, isotopic score 80, and rank score ≥25. The identification results were reviewed using the interactive HTML report and further filtered out to achieve the highest confidence in identification.

To validate the lipid annotations from both software, the retention time of the given lipid species against its Kendrick mass defect (KMD) to the hydrogen base was plotted and evaluated for possible inaccuracies in the annotation according to the detailed description of retention time mapping for lipids of different (sub)classes by Lange and Fedorova.^26^

### 2.6 Lipid Quantification

Skyline v.21.1.0.146 (MacCoss Lab)^27^ was used for the quantification of sphingolipids, phospholipids, and glycerolipids. For each lipid, the corresponding precursor ion was selected for the peak integration. The peak boundaries were delimited, manually corrected, and verified. Type I isotopic correction as well as correction for the incomplete labeling of deuterated ISTDs was applied. Quantitative values for lipid species were determined by dividing the corrected peak area for lipid species with the peak area of the used ISTD and multiplying with the concentration of the specific ISTD for each lipid class. The obtained peak areas were normalized by appropriate lipid species from SPLASH Lipidomix Mass Spec Standard (Avanti):^28^ D18:1–18:1(d9) SM for ceramides and sphingomyelins, 18:1(d7) Lyso PC for lysophosphatidylcholines, 18:1(d7) Lyso PE for lysophosphatidylethanolamines, 15:0–18:1(d7) PC for phosphatidylcholines, 15:0–18:1(d7) PE phosphatidylethanolamines, 15:0–18:1(d7) DAG for diglycerides, and 15:0–18:1(d7)-15:0 TAG for triglycerides. Missing value replacement (MVR) with half of the minimum value for each lipid species was performed to replace all zero values, which may affect the results in the multivariate analysis prior to quantification with ISTDs.

### 2.7 Data Analysis and Visualization

Lipids concentration in serum are reported in μM and all continuous data which include several biochemical measurements are presented as medians ± standard deviation (SD), whereas categorical data are presented as frequencies (*N*) with percentages (*N*%).

For the statistical analysis of the data, various softwares were used. IBM SPSS Statistics for Windows, version 26 (IBM Corp., Armonk, N.Y.), was utilized for univariate statistics. To assess the differences between the study groups, the Mann–Whitney *U* test for paired comparisons was utilized for continuous parameters, while the χ^2^ test was used for pairwise comparisons of categorical parameters. Statistical significance was set at *p* ≤ 0.01 and differential lipids are presented as median concentrations with lower and upper limits of confidence intervals (CIs) at 99%. The stringent criterion of *p* ≤ 0.01 was applied to include only lipids with the highest statistical significance.

Multivariate statistical analysis was performed with SIMCA 13.0.3 (UMETRICS AB Sweden).^29^ Data were processed by principal component analysis (PCA) and partial and orthogonal-partial least-squares discriminant analyses (PLS-DA, OPLS-DA). The quality of the models, goodness of fit in the *X* (*R*^2^*X*) and *Y* (*R*^2^*Y*) variables, and predictability (*Q*^2^) were determined by permutation and CV ANOVA analysis. Significant lipids were extracted via an *S*-plot using the absolute *p* and *p*(corr) values as a cutoff. Only features with a VIP value >1, *p*(corr) > |0.5|, and *p* value > |0.05| were considered statistically significant. Logarithmic transformation of the data and UV scaling were used in all multivariate models. Lipids with QC CV values >30% were removed. Additionally, only lipids that were found to be significant in both multivariate and univariate analyses were considered statistically significant.

To explore the predictive ability of the obtained lipidomic data set in combination with biochemical markers, a ML approach was applied. ML predictive models were produced using the double cross-validation (nested) approach. Accurate tuning of hyperparameter values was an important factor in the model performance. The F1 score and Matthew’s correlation coefficient (MCC) were used comparatively as the optimization metrics.^30^ In parallel, the receiver operating the characteristic area under the curve (ROC AUC) score was also calculated for each model. Each model was evaluated 5 times using different randomization settings in each iteration to assess the robustness of the results.

Once the optimal hyperparameters were determined, they were used by the outer loop to train the classification model, whose performance was determined using the corresponding test set. A greedy algorithm was used to identify the optimal combination of the most important features in the data set to achieve the optimal prediction result. Specifically, each prediction model was first trained using the full set of features in the data set. The features were then sorted in descending order, based on the significance coefficient assigned to them by the prediction model. Several subsets of the original data set were created using an increasing number of the most important features, ranging from 1 to 45, which were finally evaluated for the predictive performance of the models they produced.

Each predictive model was validated using the permutation test as described by Lindgren et al.^31^ A set of 100 permuted response variables was created by randomly reordering the entry values of the original response variable. The permuted response variables were used one at a time to create the corresponding prediction models. In each run, the evaluation metric values were calculated and recorded. Finally, the results of the permuted models were compared with those of the reference model, which was produced using the intact response variable.

In addition, especially for the final predictive model, learning curves were examined, which provide a dynamic graphical representation of how a model performance evolves in response to changes in the size of the training data set (model complexity).^32^

## 3 Results

**Figure 1:**
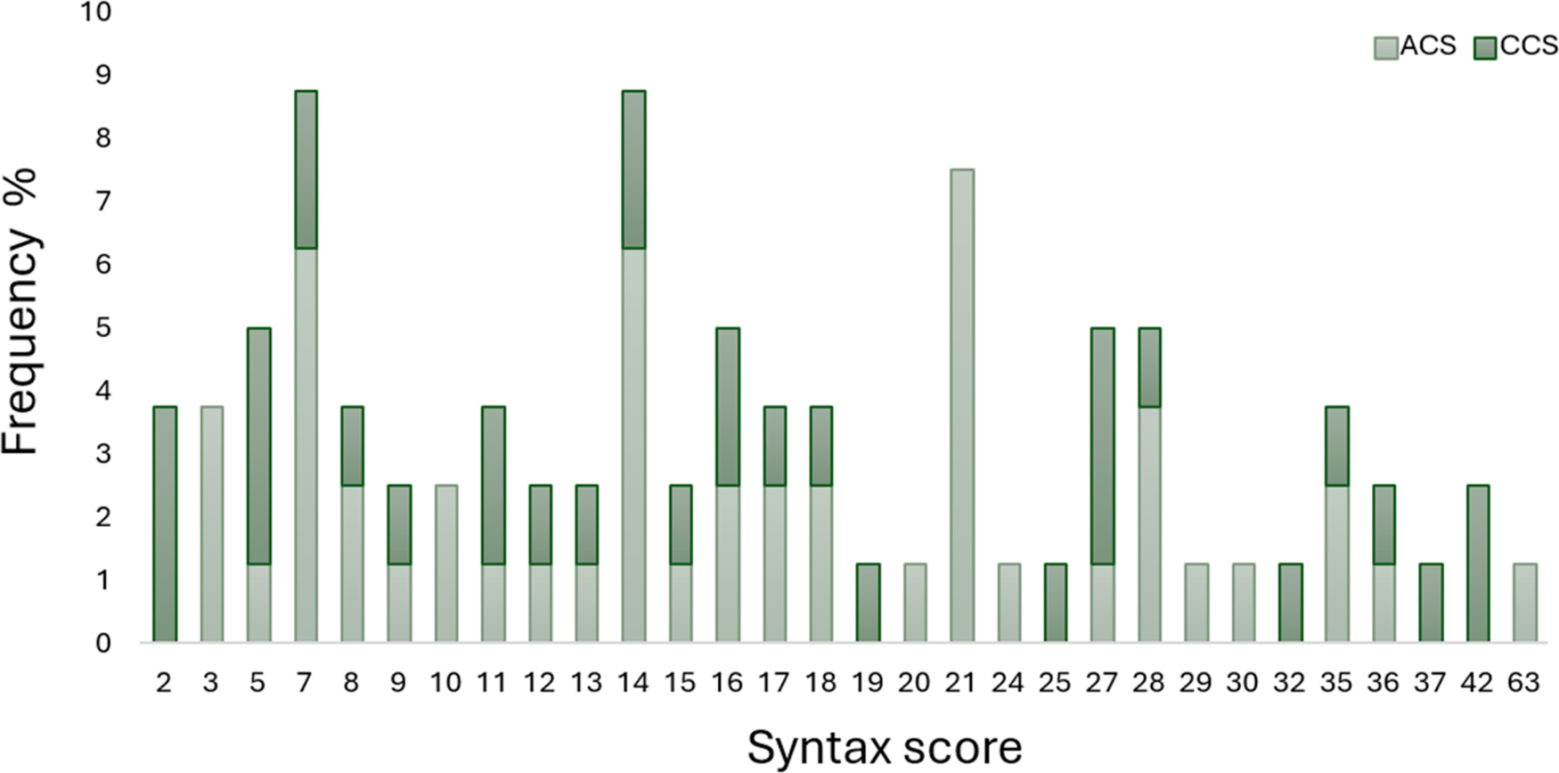
Distribution of SS values within the CAD groups (*N* = 80).

In the present study, the lipid profile was investigated in a subgroup of 146 patients enrolled in the CorLipid study by untargeted liquid chromatography with tandem mass spectrometry (LC-MS/MS) analysis. Of these, 32.9% (*N* = 48) developed acute coronary syndrome and 67.1% (*N* = 98) suffered from chronic coronary syndrome. According to the calculated SS score, 54.8% of patients (*N* = 80) had obstructive CAD (SS > 0) and 45.2% (*N* = 66) of patients had nonobstructive CAD (SS = 0). As shown in Figure 1, the distribution of SS values in the studied population is demonstrated. Demographic and clinical characteristics for the two groups of patients are described in Table 1. The median age of the participants was 62 years and 75.3% of them were men. There were no statistically significant differences between the two groups in terms of age, BMI, gender, and other risk factors such as dyslipidemia, hypertension, diabetes mellitus type II, and smoking. Comorbidities associated with CAD, including previous stroke, chronic kidney failure, peripheral vascular disease, and chronic obstructive pulmonary disease were investigated as well, but no statistically significant differences were found between the groups. Additionally, the medication of the patients was recorded. Biochemical markers were tested with a simple blood analysis. Total cholesterol, total triglycerides, high-density lipoprotein (HDL-c), low-density lipoprotein (LDL-c), troponin, lactate dehydrogenase (LDH), glutamic-oxaloacetic transaminase (SGOT), and creatine phosphokinase (CPK) showed a statistically significant difference between the studied groups.

**Table 1:** Baseline Characteristics of the Patients’ Cohort (*N* = 146)^a^

	SYNTAX groups					
		SS = 0 (*N* = 66)		SS > 0 (*N* = 80)		
		median	SD	median	SD	*p* value
age (years)		62	10.9	62	10.1	0.894
BMI (kg/m^2^)		27.7	3.67	27.8	4.86	0.670
SYNTAX score		0	0	15.5	11.3	
		*N*	*N*%	*N*	*N*%	*p* value
gender	female	22	33.3	16	20.0	0.067
	male	44	66.7	64	80.0	
CAD groups	CCS	65	98.4	33	41.2	<0.001
	ACS	1	1.52	47	58.7	
dyslipidemia	no	45	68.2	60	75.0	0.361
	yes	21	31.8	20	25.0	
hypertension	no	37	56.1	36	45.0	0.937
	yes	29	43.9	29	55.0	
diabetes mellitus	no	51	77.3	60	75.0	0.748
	yes	15	22.7	20	25.0	
family history	no	57	86.4	62	77.5	0.169
	yes	9	13.6	18	22.5	
smoking	no	41	62.1	42	52.5	0.242
	yes	25	37.9	38	47.5	
chronic kidney failure	no	65	98.5	77	96.3	0.280
	yes	1	1.50	3	3.80	
peripheral vascular disease	no	62	93.9	78	97.5	0.498
	yes	4	6.10	2	2.50	
chronic pulmonary obstructive disease	no	63	95.5	78	97.5	0.797
	yes	3	4.50	2	2.50	
previous stroke	no	65	98.5	77	96.3	0.412
	yes	1	1.50	3	3.80	
	Medication					
clopidogrel	no	60	90.9	63	78.8	0.062
	yes	6	9.10	16	20.0	
anticoagulants	no	56	84.8	72	90.0	0.241
	yes	10	15.2	7	8.80	
b-blockers	no	39	59.1	49	61.3	0.718
	yes	27	40.9	30	37.5	
angiotensin-converting enzyme inhibitors	no	60	90.9	65	81.3	0.133
	yes	6	9.10	14	17.5	
statins	no	44	66.7	55	68.8	0.703
	yes	22	33.3	24	30.0	
calcium channel blockers	no	60	90.9	70	87.5	0.650
	yes	6	9.10	14	17.5	
angiotensin receptor blockers	no	51	77.3	59	73.8	0.716
	yes	15	22.7	20	25.0	
diuretics	no	57	86.4	63	78.8	0.293
	yes	9	13.6	16	20.0	
	Biochemical Markers					
		median	SD	median	SD	
glucose mg/dL		101	24.0	104	45.4	0.073
creatinine mg/dL		0.85	1.19	0.92	0.26	0.167
cholesterol (total) mg/dL		178	26.7	208	26.6	<0.001
triglycerides mg/dL		104	42.9	139	65.3	<0.001
HDL-c mg/dL		45.0	12.7	40.5	9.97	0.004
LDL-c mg/dL		111	22.9	140	23.3	<0.001
TnThs ng/L		12.0	23.6	134	2064	<0.001
SGOT U/L		19.0	37.7	27.0	85.9	<0.001
SGPT U/L		18.5	78.2	24.0	17.0	0.052
LDH U/L		209	75.0	311	351	<0.001
CPK U/L		84.0	79.7	178	801	<0.001
galectin-3 ng/mL		8.72	5.42	9.60	6.11	0.307

a Categorical parameters are represented by their respective counts (*N*) and the corresponding relative frequencies (*N*%), while continuous parameters are represented in terms of their median values and standard deviations (medians, SD). The Mann–Whitney *U* test was conducted to assess the statistical significance of the comparison between the two distinct SYNTAX score groups (SS = 0 and SS > 0) for continuous parameters, while Chi-square was utilized for categorical parameters. The threshold for statistical significance was set at *p* < 0.01. Abbreviations: BMI: body mass index, HDL-c: high-density lipoprotein, LDL-c: low-density lipoprotein, TnThs: HS-troponin T, SGOT: glutamic-oxaloacetic transaminase, SGPT: glutamic pyruvic transaminase, LDH: lactate dehydrogenase, CPK: creatine phosphokinase, and SD: standard deviation.

**Figure 2:**
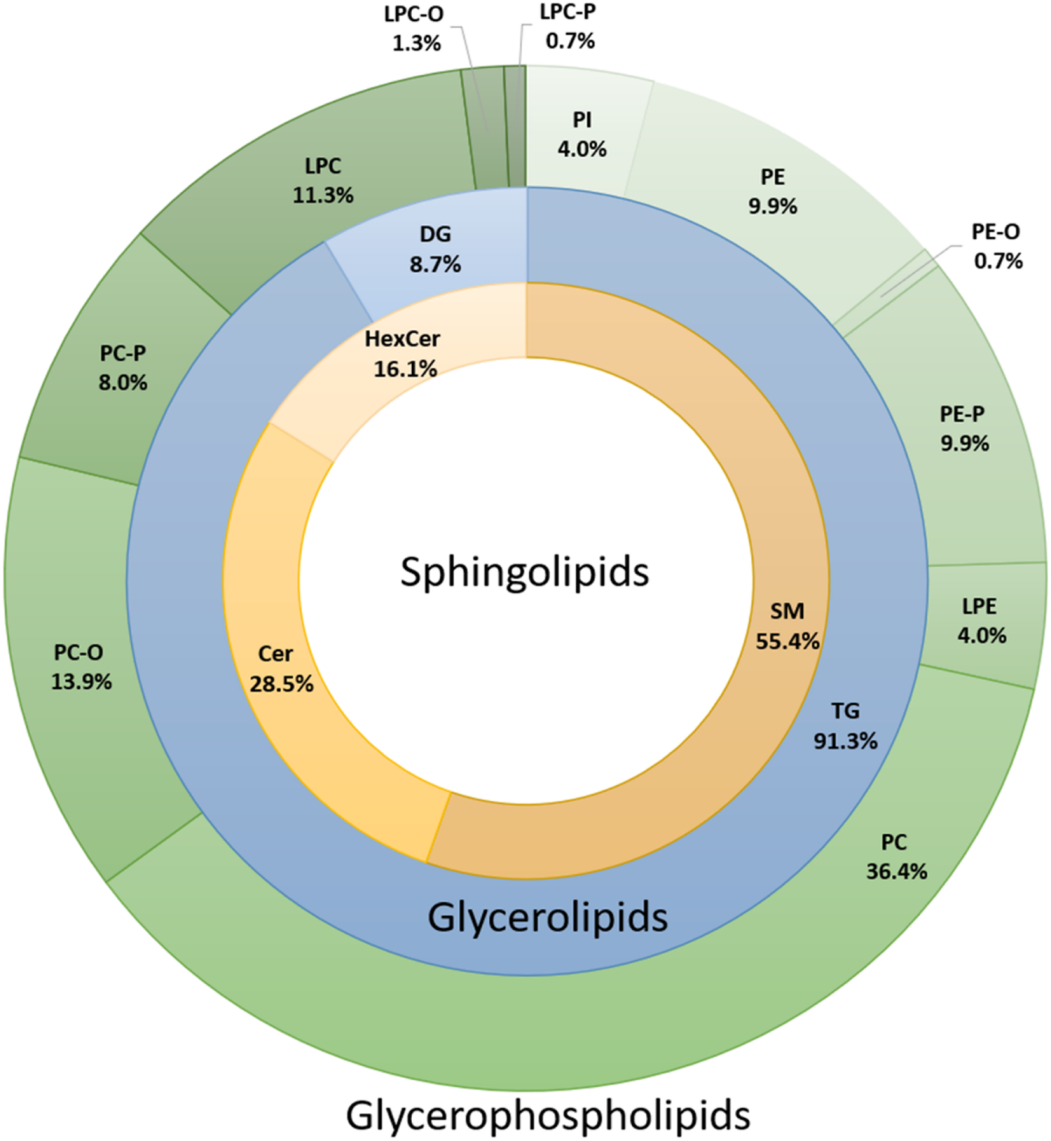
Classification of the lipid species quantified in the serum of patients with CAD (*N* = 146).

In total, 517 lipid species could be identified by the applied strategy (Table S1), from which 288 lipid species were quantified and the concentrations were expressed in μM. The validity of the analytical data was assessed by analyzing the QC samples. The PCA score plot, which includes all samples and QC samples, indicated satisfactory analytical precision and it is presented in Figure S1. Furthermore, lipids with CV % values greater than 30% in QC samples were excluded from further processing. Among the quantified lipid species, glycerophospholipids constituted 52.4%, glycerolipids accounted for 28.1%, and sphingolipids comprised 19.4%. A schematic diagram of the most representative lipid species quantified in the patients’ serum is presented in Figure 2. The detailed table with all annotations including the molecular formula, exact masses, and retention time data is provided in Table S1.

### 3.1 Correlation of the Lipid Profile with the SYNTAX Score in Patients with Obstructive CAD

**Figure 3:**
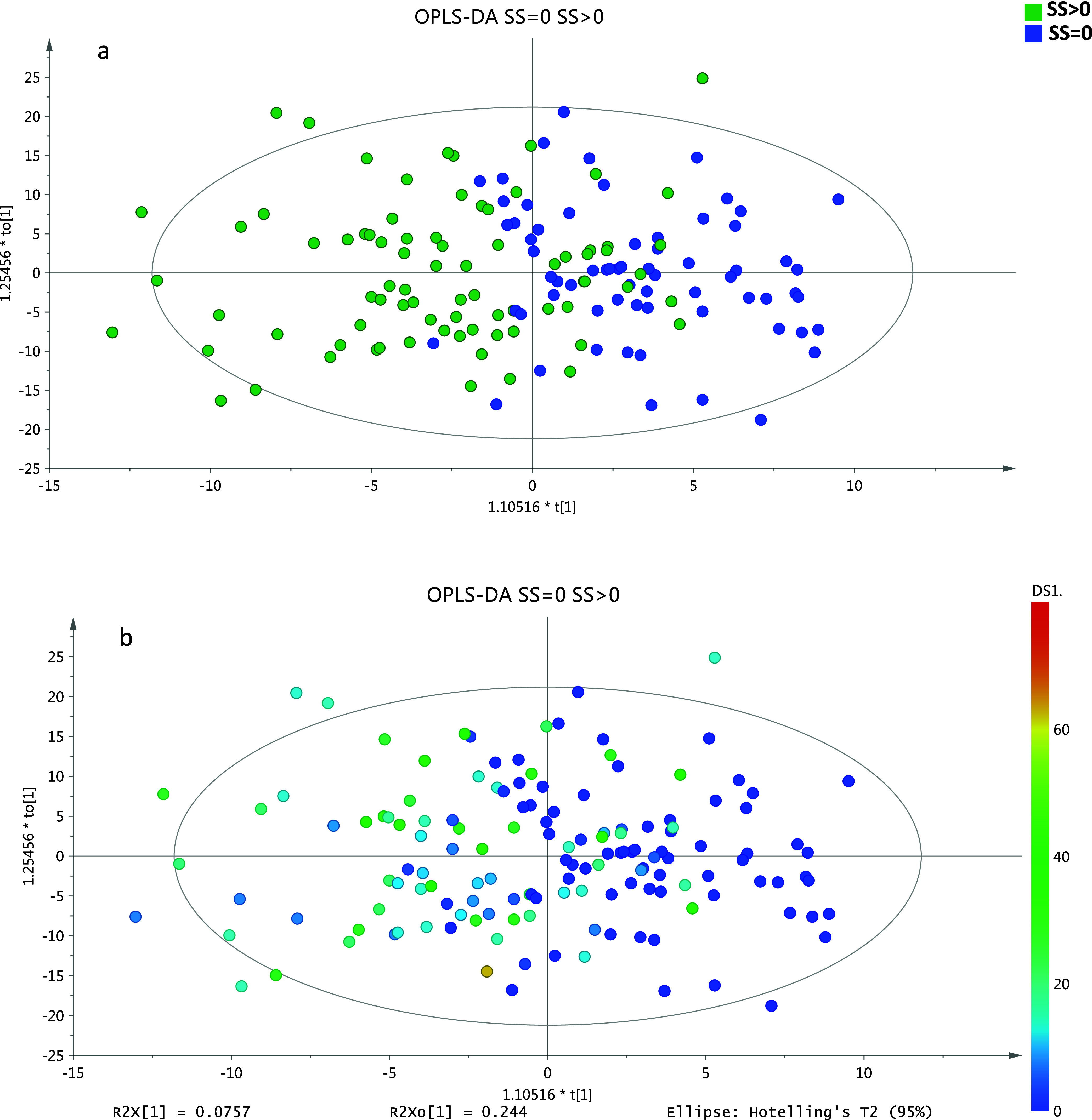
(a) OPLS–DA score plot showing the classification of individuals with SS = 0 (*N* = 66) and patients with SS > 0 (*N* = 80) based on the serum lipidome. (b) OPLS–DA score plot showing the classification of individuals with SS = 0 (*N* = 66) and patients with SS > 0 (*N* = 80) based on the serum lipidome, where patients with SS > 0 are depicted in a different color scale relative to the SS value. Logarithmic transformation of the data and UV scaling were used in all multivariate models (*R*^2^*X* 0.385, *R*^2^*Y* 0.378, *Q*^2^ 0.155, CV anova *p* = 0.000568).

The quantified lipids underwent further analysis, and the discerned patterns may suggest potential associations with obstructive CAD. Multivariate statistical analysis models were constructed based on serum lipid levels for the two SS groups: SS = 0 and SS > 0. As demonstrated in the OPLS-DA score plot in Figure 3a, the group of patients diagnosed with obstructive CAD exhibit a distinct serum lipid profile, which discriminates them from the SS = 0 group. The characteristics of the model describing its reliability are *R*^2^*X* 0.385, *R*^2^*Y* 0.378, and *Q*^2^ 0.155, while the value of *p* = 0.000568 refers to the validation of the model by CV ANOVA analysis. The patients’ diverse levels of obstructive CAD, as indicated by their SS values, are observable in the trend depicted in the OPLS-DA score plot, as shown in Figure 3b. Individuals with SS = 0 values are presented in blue dots and patients with SS > 0 are demonstrated in a differenced green color scale, in which lighter green dots correspond to higher SS values. As shown in Table 2, the statistically significant differentiated lipids are presented as derived from the multivariate analysis along with their p values from the Mann–Whitney *U* test, median concentrations, and the lower and upper bounds of the 99% confidence intervals (CIs). Upregulated lipid species in the SS > 0 group comprised of the sphingolipid class, namely, Cer 18:1;O2_16:0, Cer 18:1;O2_18:0, Cer 18:1;O2_24:1, SM 36:0;O2, and SM 36:1;O2. Two phosphatidylcholines PC 32:0 and PC 34:1, two diglycerides DG 34:1 and DG 36:2, and one triglyceride TG 52:1 played an important role in the discrimination of the two groups, as they were found elevated in the serum of patients with SS > 0. The initial discrimination of the two studied groups, observed in the multivariate analysis, was further evaluated with the ML approach where biochemical parameters were considered for adjustment.

**Table 2:** Lipids with Significantly Different Levels across SYNTAX Score Groups, SS = 0 (*N* = 66) and SS > 0 (*N* = 80) (Median Concentrations are Expressed in μM)

			SS = 0			SS > 0		
lipids	Mann–Whitney *p* value <0.01	VIP	median	99% lower CI	99% upper CI	median	99% lower CI	99% upper CI
Cer 18:1;O2_16:0	6.86 × 10^–4^	1.63	0.29	0.26	0.30	0.32	0.30	0.34
Cer 18:1;O2_18:0	3.81 × 10^–5^	1.96	0.13	0.11	0.17	0.18	0.15	0.21
Cer 18:1;O2_24:1	1.07 × 10^–5^	1.63	2.80	2.59	2.99	3.31	3.06	3.65
SM 36:0;O2	8.98 × 10^–3^	1.59	1.96	1.67	2.67	2.73	2.16	3.30
SM 36:1;O2	8.47 × 10^–6^	1.99	43.9	40.0	50.0	54.2	49.5	59.7
PC 32:0	3.14 × 10^–4^	1.64	39.4	35.9	44.4	45.3	42.2	49.5
PC 34:1	1.98 × 10^–4^	1.61	508	472	549	582	554	610
DG 34:1	1.27 × 10^–4^	1.81	9.55	7.62	11.4	14.0	12.2	15.7
DG 36:2	1.95 × 10^–4^	1.63	28.4	24.2	32.7	41.1	34.3	45.8
TG 52:1	7.54 × 10^–3^	1.62	42.0	26.0	54.9	60.1	51.5	74.5

### 3.2 Machine Learning Modeling for Obstructive CAD Prediction

Τo investigate the capability of the information obtained from lipidomic data on the discrimination and prediction of obstructive CAD, a ML approach was employed. A series of predictive ML models were built and evaluated, aiming to classify the patients into 2 categories (binary classification), those with SS > 0 and those with SS = 0.

The best performance was achieved by combining the XGBoost algorithm with the F1 score as the optimization metric. The initial model included only the data from lipidomic analysis (unadjusted model) giving acceptable values of MCC: 0.177, AUC: 0.647 (95%CI: 0.616–0.679), sensitivity: 92.5%, specificity: 18.2%, PPV: 57.8%, and NPV: 66.7%. The next step was to adjust the model including values of biochemical markers, namely, glucose, creatinine, total cholesterol, total triglycerides, HDL-c, LDL-c, SGOT, SGPT, LDH, CPK, and galectin-3, attaining the optimal performance [MCC: 0.653, AUC ROC: 0.800 (95%CI: 0.772–0.827), sensitivity: 100%, specificity: 57.6%, PPV: 74.1%, NPV: 100%].

**Figure 4:**
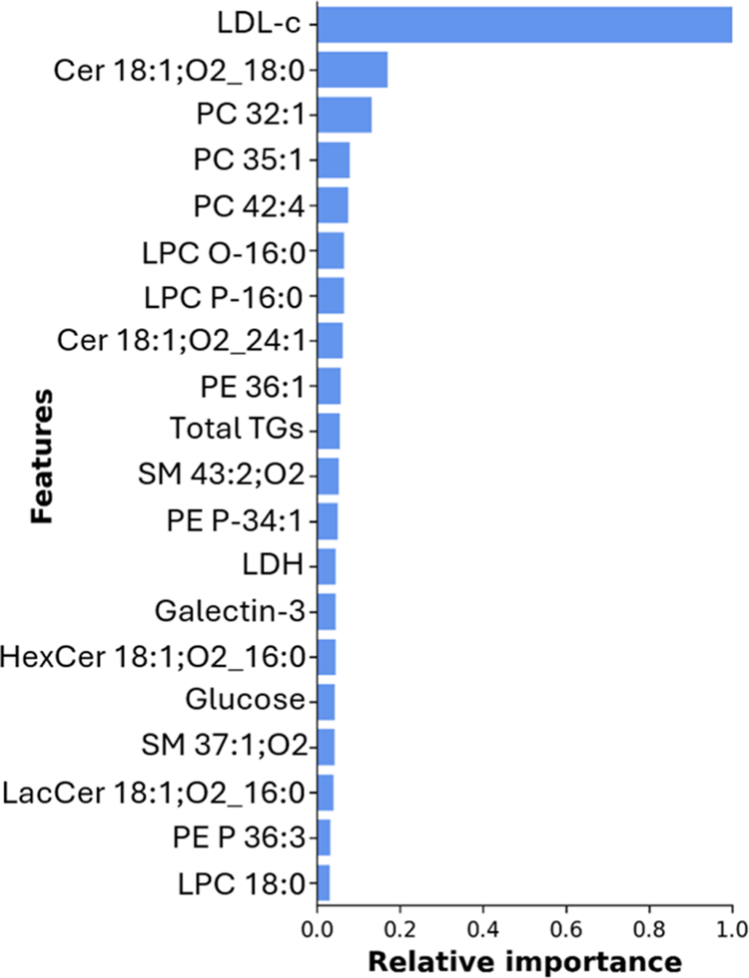
Bar plot representing the relative importance of the top 20 most important markers of the data set.

The XGBoost algorithm was able to rank the data set variables in terms of their importance in achieving the classification. Based on the model, the significant lipids and biochemical markers were recognized. As shown in Figure 4, the 20 most important lipids and biochemical markers of the data set are presented with their relative importance. When the greedy algorithm was employed to assess the impact of the feature number on the performance of the model (reference model), the optimal outcome [MCC: 0.688, ROC AUC score: 0.888 (95%CI: 0.880–0.896), sensitivity: 100%, specificity: 62.1% PPV: 76.1%, NPV: 100%] was achieved utilizing the first 17 lipids and biochemical markers, namely, LPC O-16:0, LPC P-16:0, Cer 18:1;O2_18:0, Cer 18:1;O2_24:1, HexCer 18:1;O2_16:0, SM 37:1;O2, SM 43:2;O2, PC 32:1, PE P-34:1, PE P-34:1, PE 36:1, PC 35:1, total triglycerides, glucose, LDH, LDL-c, and galectin-3. The performance of the optimized model was significantly improved compared to the reference model, with a net reclassification improvement (NRI) value of 0.070 (95%CI: 0.030–0.101) and an integrated discrimination index (IDI) value of 0.093 (95%CI: 0.062–0.114).

**Figure 5:**
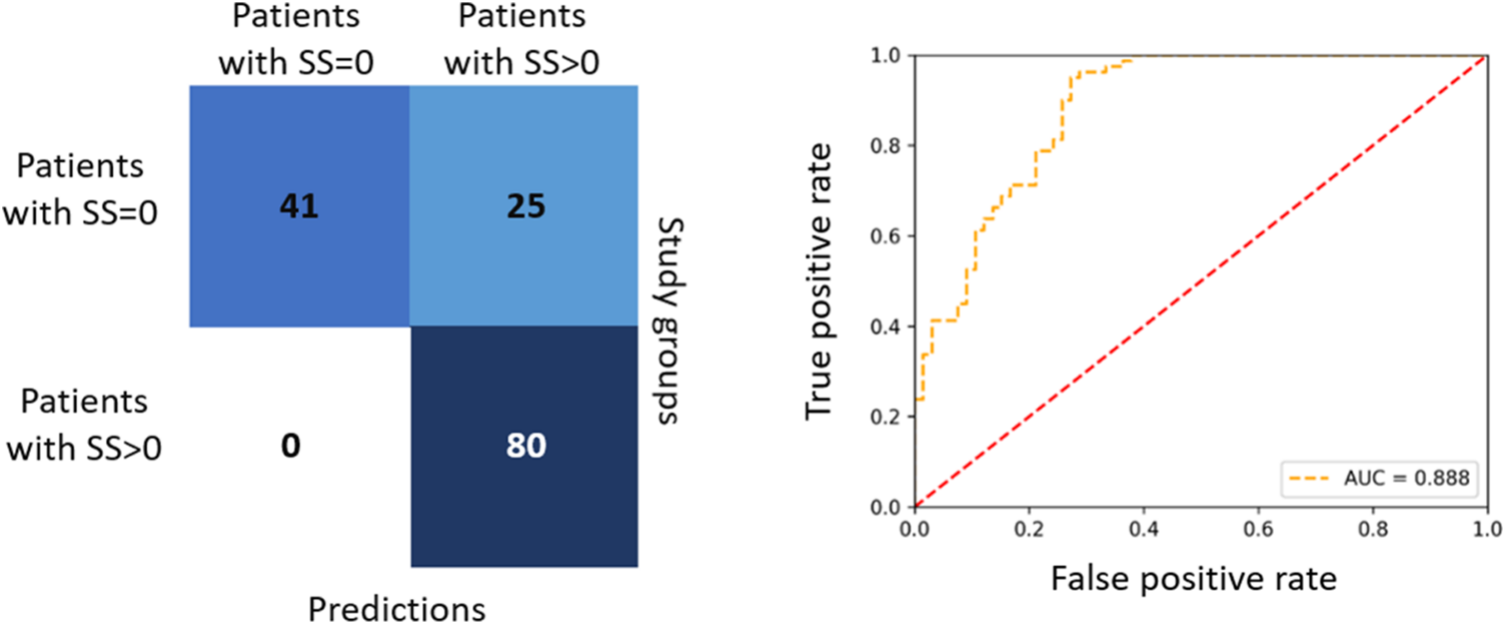
(a) Confusion matrix with the results of the patients’ classification achieved by the XGBoost algorithm. (b) ROC AUC plot describing the performance of the optimal model.

**Figure 6:**
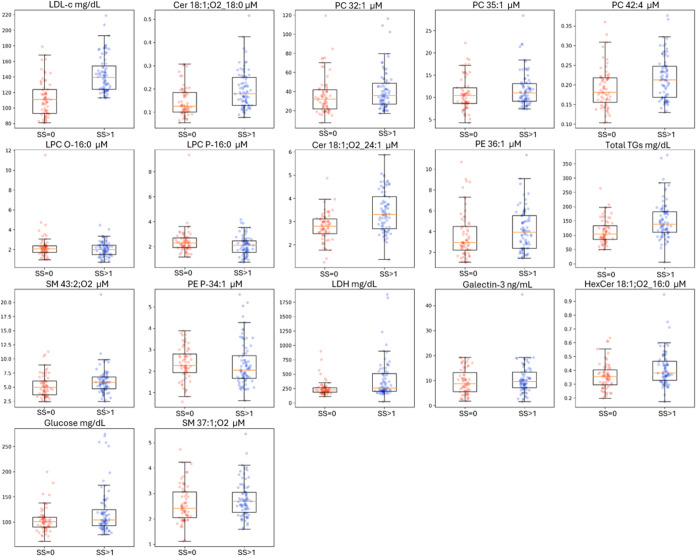
Boxplots demonstrating the distribution of the biochemical markers and lipids in SS = 0 (*N* = 66) and SS > 0 (*N* = 80) groups.

As shown in Figure 5a, the confusion matrix with the results of the patient classification achieved by the model is presented, while as shown in Figure 5b, the corresponding ROC AUC plot is demonstrated. The model was able to classify 41 out of 66 patients (62.1%) in the group without obstructive CAD (SS = 0), while all patients with SS > 0 (80/80, 100%) were correctly classified to the group with obstructive CAD. However, in imbalanced data sets, it is essential to use evaluation metrics such as Matthew’s correlation coefficient (MCC), the area under the precision-recall curve (AUC-PR), balanced accuracy, Cohen’s kappa, and geometric mean (G-Mean), along with threshold decision adjustments to prioritize the minority class. These metrics provide a more accurate assessment of the model performance. Additionally, employing techniques such as resampling (oversampling the minority class or undersampling the majority class), utilizing algorithms tailored for imbalanced data, and applying cost-sensitive learning can significantly enhance the model’s ability to accurately predict minority class instances.^33−36^Table 3 summarizes the biochemical parameters and lipids which are included in the optimized model, while the boxplots shown in Figure 6 illustrate the distribution of the values of the specific markers between the two groups of SS.

**Table 3:** Significant Markers Which Enhanced the Classification of Patients (*N* = 146) into Two SS Groups, SS = 0 (*N* = 66) and SS > 0 (*N* = 80), as Derived from the XGBoost Algorithm

		SS = 0			SS > 0		
significant markers from the algorithm	Mann–Whitney *Up* value	median	99% lower CI	99% upper CI	median	99% lower CI	99% upper CI
LPC O-16:0 μM	4.65 × 10^–1^	2.09	1.93	2.25	2.01	1.84	2.20
LPC P-16:0 μM	2.00 × 10^–2^	2.29	2.08	2.43	2.11	1.79	2.22
Cer 18:1;O2_18:0 μM	<1.00 × 10^–4^	0.13	0.11	0.15	0.18	0.16	0.20
Cer 18:1;O2_24:1 μM	<1.00 × 10^–4^	2.80	2.66	2.98	3.31	3.12	3.57
HexCer 18:1;O2_16:0 μM	3.00 × 10^–3^	0.36	0.33	0.37	0.38	0.36	0.43
SM 37:1;O2 μM	5.90 × 10^–2^	2.41	2.25	2.62	2.69	2.53	2.79
SM 43:2;O2 μM	1.10 × 10^–2^	4.95	4.37	5.44	5.82	5.38	5.95
PC 32:1 μM	4.70 × 10^–2^	32.0	26.7	35.7	35.7	31.6	40.4
glucose mg/dL	7.04 × 10^–2^	101	94.0	106	104	100	118
total triglycerides mg/dL	<1.00 × 10^–4^	103	89.0	124	138	120	169
LDL-c mg/dL	<1.00 × 10^–4^	111	105	118	139	128	146
LDH mg/dL	1.70 × 10^–3^	209	187	231	311	223	485
galectin-3 ng/mL	3.53 × 10^–1^	8.70	6.00	10.9	9.40	8.30	10.5
PE P-34:1 μM	3.40 × 10^–2^	2.26	2.13	2.58	2.05	1.82	2.25
PE 36:1 μM	1.84 × 10^–1^	2.92	2.58	3.41	3.90	3.26	4.35
PC 35:1 μM	9.30 × 10^–2^	10.4	8.97	10.8	11.0	9.84	11.8
PC 42:4 μM	3.00 × 10^–3^	0.18	0.17	0.20	0.21	0.20	0.23

The optimized model was validated using the permutation test, while the corresponding learning curves indicate a lack of overfitting. The validation curve gradually increases, approaching the training curve and finally reaching a plateau, as demonstrated in Supporting Figure S2.

## 4 Discussion

Herein, we discuss the outcomes derived from a lipidomic-based analysis of serum samples from 146 participants of the CorLipid trial with suspected CAD undergoing invasive coronary angiography.^37^ We investigated changes in lipidomes to identify lipids potentially associated with the phenotype and complexity of CAD. Using this information, we explored the utility of 288 quantified serum lipid species for developing a ML predictive algorithm to assess CAD severity.

Successful prediction was achieved using only lipidomic data and markers from a simple biochemical test, including glucose, LDL-c, and LDH. Galectin-3 was integrated into the model to augment its predictive strength. The predictive performance of the initial model, including only lipidomic data [MCC: 0.177, AUC: 0.647 (95%CI: 0.616–0.679), sensitivity: 92.5%, specificity: 18.2%, PPV: 57.8%, and NPV: 66.7%], was further improved when biochemical parameters were added. According to our final ML model, a panel of 17 lipid biomarkers that play a pivotal role in cardiomyocyte metabolism have demonstrated their ability to accurately predict the absence or presence of obstructive CAD with 100% sensitivity and 62.1% specificity. The included biomolecules encompass five sphingolipids (ceramides, neutral glycosphingolipids, and phosphosphingolipids) and seven glycerophospholipids, namely, LPC O-16:0, LPC P-16:0, Cer 18:1;O2_18:0, Cer 18:1;O2_24:1, HexCer 18:1;O2_16:0, SM 37:1;O2, SM 43:2;O2; PC 32:1, PE P-34:1, PE P-34:1, PE 36:1, PC 35:1, total triglycerides, glucose, LDH, LDL-c, and galectin-3. In a diagnostic clinical model, the importance of increased sensitivity versus increased specificity depends on the context and the clinical consequences of false positives versus false negatives. In obstructive CAD prediction, and if the proposed lipidomic model is confirmed in larger samples and similar populations, a two-step testing process could be used: a highly sensitive initial test (the proposed lipidomic model) followed by a highly specific confirmatory test (invasive coronary angiography, ICA). Thereby, the lipidomic model could have applicability as a preliminary test for precisely ruling out CAD diagnosis in patients with intermediate or low clinical likelihood of CAD, either as a stand-alone test or as a complementary diagnostic blood test to coronary CT angiography. Using this approach, clinicians could effectively rule out CAD or reclassify patients from intermediate/low to high post-test probability of CAD, where ICA should be performed. Nowadays, mass spectrometry is used in almost all areas of laboratory medicine and dedicated methods for the quantifications of the specific lipid markers can be applied in mass spectrometry instrumentation in the clinical setting.

Up to now, the integration of clinical risk factors has resulted in the development of clinical risk algorithms, such as the Framingham risk score, systematic coronary risk evaluation 2 (SCORE2), and second manifestations of arterial disease 2 (SMART2). These algorithms, however, are predominantly based on traditional risk factors, including age, smoking, hypertension, diabetes, and hypercholesterolemia. Consequently, they are limited in their ability to incorporate the multitude of pathophysiological processes that contribute to the onset and progression of CAD.^38−40^ The novelty of our study lies in the utilization of circulating lipid species in conjunction with established risk factors to enhance the sensitivity of patient classification.

Some of the aforementioned lipids and biochemical markers are widely acknowledged as risk factors; for instance, LDH serves as a pathologic marker for various diseases, including myocardial ischemia.^41^ LDL-c is widely associated with CAD, given its role in transporting cholesterol and triglycerides from the liver to cardiomyocytes. This transport is essential for building cell membranes and facilitating various cellular processes. However, an excess of this process results in the packing of fatty acids into a glycerol backbone, forming triglycerides. This overloading leads to saturation and, subsequently, to lipid accumulation. Regions with compromised blood vessel integrity lead to the development of atherosclerotic plaques. Lipids bind to apolipoproteins, penetrate vessel walls, and initiate inflammation and foam cell formation. LDL-c aggregation results in the accumulation of cholesteryl ester-rich droplets, the release of matrix metalloproteinase-7, and the activation of T-cells. Galectin-3 is closely associated with CAD,^42−44^ influencing macrophage differentiation, foam cell formation, and vascular smooth muscle cell proliferation and migration, contributing to the initiation and progression of atherosclerosis.

Besides these, ceramides, the key representatives of the sphingolipid class, seem to have a significant role in ACS and myocardial infarction, as plaque rupture or erosion frequently depends on plaque composition and vulnerability.^45^ Under pathological conditions characterized by elevated levels of sphingomyelins and ceramides, susceptibility increases, and these sphingolipids, particularly ceramides, become extremely deleterious for the heart.^45^ Previous studies have consistently demonstrated the significant predictive value of ceramides, ceramide ratios, and specific ceramide species, particularly the ratio of Cer 18:1;O2/24:1/Cer 18:1;O2/24:0, Cer 18:1;O2/14:0, and Cer 18:1;O2/22:0, as independent indicators of ACS. This predictive power is notably enhanced when these ceramides are combined with high-sensitive troponin and traditional risk factors.^46^

Furthermore, a recent study comprising 248 STEMI patients reaffirmed the independent association of elevated levels of Cer 18:1;O2/16:0, Cer 18:1;O2/18:0, and Cer 18:1;O2/24:1 with increased SS values.^47^ In a related study encompassing 553 patients, the severity of coronary artery stenosis was linked to a high Cer 18:1;O2/24:1 to Cer 18:1;O2/24:0 ratio.^48^ Recently, our group investigated the relationship between serum ceramide levels and the micro-CT-quantified thrombotic burden in 38 STEMI patients, indicating that Cer 18:1;O2/24:0 and Cer 18:1;O2/24:1 could predict a higher thrombus burden.^37^ These findings underscore the significance of these specific ceramides in assessing CAD severity.

Our analyses yielded that elevated levels of Cer 18:1;O2_16:0, Cer 18:1;O2_18:0, Cer 18:1;O2_24:1, SM 34:1;O2, SM 36:0;O2, SM 36:1;O2, SM 36:2;O2, SM 38:0;O2, SM 38:1;O2, SM 40:1;O2, SM 42:1;O2, and SM 42:2;O2 might be encountered in patients with obstructive CAD compared to those without. Notably, Cer 18:1;O2_18:0, Cer 18:1;O2_24:1, and GlcCer 18:1;O2_16:0 emerged as particularly significant biomarkers, identified by the XGBoost algorithm, with a high predictive value for SS > 0. Long-chain and very-long-chain ceramides have also emerged as potential markers for increased mortality risk in ACS, surpassing the limitations of conventional assessment tools.^49^

PCs constitute a class of phospholipids distinguished by diverse fatty acid chains tethered to the glycerol backbone, playing a pivotal role in signaling, inflammation, and providing energy substrates. Serving as essential energy storage depots, these molecules undergo enzymatic degradation, cleaving the ester bond between the glycerol backbone and releasing fatty acids, alongside LPC. Fatty acids are channeled for β-oxidation, while the glycerol backbone contributes to glycolytic intermediates. Glycerophospholipid hydrolysis in the cardiomyocyte membrane has been associated with the pathogenesis of myocardial infarction changes since approximately 40% of human cardiomyocytes are composed of PCs. Alterations in fatty acid β-oxidation, as well as modifications induced by catabolic enzymes like phospholipases, compromise important structural lipids of the heart, influencing heart function during ischemia/reperfusion.^50^

The metabolism of glycerophospholipids, α-linoleic acid, and sphingolipids exhibited robust correlations with STEMI, unveiling concealed metabolic disruptions. This revelation stems from a comprehensive study that delved into the phenotypic characterization of STEMI patients compared to their healthy counterparts.^51^ A targeted ultra performance liquid chromatography with tandem mass spectrometry (UPLC-MS/MS) analysis revealed lower concentrations of 15 plasma PCs, while a FIA-MS analysis indicated higher concentrations of 16 plasma PCs.^52,53^ PC reduction was linked to an inhibition of their synthesis during hypoxia or ischemia, hinting at a depletion of ATP and CTP. This depletion may contribute to the cardiac struggling for optimal function.^52,53^ On the other hand, elevated PC levels of phosphatidylcholines can be explained as well. There is evidence supporting a correlation between the selection of phospholipase A for specific substrates and the mechanisms of enzyme activation.^51,54^ In an intriguing experimental model, a study observed a remarkable, over 400% increase in the activity of membrane-associated calcium-independent plasmalogen-selective phospholipase A2 during a 2 min global ischemia. This activity peaked after 5 min and sustained throughout the examined 60 min period.^51,55^ Given the above, a plausible explanation is that following reperfusion and beyond the phases of injury and inflammation, there could be a surge in phospholipid biosynthesis, reflecting ongoing repair processes.

In our study, similar to the referenced research above, we observed elevated levels of PC 32:1, PC 35:1, PC 42:4, and LPC O-16:0, which were identified as predictors of obstructive CAD (SS > 0) from the constructed ML algorithm.

Recent findings from two cohorts highlighted that among the 105 studied metabolites, serum concentrations of sphingomyelins, diacyl-phosphatidylcholines, and acyl-alkyl-phosphatidylcholines were associated with the risk of STEMI.^56^ Alterations in sphingomyelin and PC metabolism, particularly involving metabolites from the arachidonic acid pathway, were independently linked to the risk of myocardial infarction in healthy adults.^56^ In our study, the XGBoost algorithm pinpointed PE 36:1 and PE P-34:1 as predictive markers for obstructive CAD. Plasmalogens, fundamental glycerophospholipids vital for the cell membrane structure, showcase unique features. Their distinction lies in the vinyl-ether group at the *sn-1* position of l-glycerol, setting them apart from the majority of membrane glycerophospholipids with ester functions at both *sn-1* and *sn-2* positions.^57^ While we acknowledge their role in CAD, the precise mechanisms remain elusive.^58^ Reduced plasma levels of PC P-33:1, PC P-33:2, PC P-33:3, and PC P-35:3 have been noted in CAD patients. A common scenario is emerging, where mitochondrial dysfunction triggers oxidative stress, depleting plasmalogens and fostering chronic inflammation.^47,58^ Consequently, there are insufficient plasmalogens to scavenge various reactive oxygen species. Plasmalogen depletion, caused by heightened oxidative stress, can result in cell membrane damage and subsequent tissue damage. This depletion is closely connected to cardiovascular disease, underscoring the significance of assessing plasmalogen levels. This may elucidate the higher concentrations of plasmalogens in cardiac tissue, strategically positioned subcellularly and extracellularly near oxidizable substrates.^51^ Nevertheless, the study is limited by the relatively small sample size of 146 participants, representing only a subset of the larger CorLipid cohort. Additionally, challenges in assessing fasting periods were encountered in some cases of STEMI, who underwent urgent coronary angiography at the time of their arrival at the emergency department. A robust validation process involving a sizable and diverse cohort across multiple centers is crucial to confirm the diagnostic potential of this panel of biomarkers. Finally, considerations related to repeatability and cost-effectiveness will be essential for practical implementation in routine clinical settings.

## 5 Conclusions

Given the high-dimensional nature of lipidomic data and the multitude of lipids in conjunction with clinical markers, traditional regression models are often deemed ineffective. Our study demonstrated the potential of advanced ML approaches for unraveling the intricate lipidomic interactions for utilizing a large pool of biomarkers for risk stratification and for shedding light on their role in CAD. The precision of the obtained results, the powerful diagnostic performance of the generated model, and the clinical meaningfulness of our findings add to the existing body of evidence and underscore the need for similar investigation efforts.

## Data Availability

The raw data are publicly accessible in the Center for Computational Mass Spectrometry’s MassIVE repository under the identifier MSV000095097, titled “Lipidomic-based Algorithms Can Enhance Prediction of Obstructive Coronary Artery Disease.″

## Supporting Information Available

PCA score plot (Figure S1); optimized model validation tests (Figure S2); and summary of all identified lipids in the blood serum of patients with and without obstructive CAD (*N* = 146) (Table S1) (PDF)

## Notes

**Ethics Approval** Every trial process was carried out in accordance with the International Conference on Harmonization Guidelines for Good Clinical Practice and the Helsinki Declaration’s guiding principles.

## Notes

**Consent to Participate** Every patient attested a written informed consent before being enrolled in the study.

## Notes

**Consent for Publication** The authors consented to publish the content of this original report.
