# Supplementary material for: Lipidomic-Based Algorithms Can Enhance Prediction of Obstructive Coronary Artery Disease
Source: J Proteome Res. 2024 Jul 15;23(8):3598–611. doi: 10.1021/acs.jproteome.4c00249 (PMC11301671; doi:10.1021/acs.jproteome.4c00249)
Supplement: Supplementary file 1 — pr4c00249_si_001.pdf [file pr4c00249_si_001.pdf]

Lipidomics-based algorithms can enhance prediction of obstructive coronary artery disease

Thomai Mouskeftara[1,2], Olga Deda[1,2], Theodoros Liapikos[2], Eleftherios Panteris[2], Efstratios Karagiannidis[3], Andreas S Papazoglou[4], Helen Gika[1,2*]

[1] Laboratory of Forensic Medicine and Toxicology, School of Medicine, Aristotle University of Thessaloniki, 54124, Thessaloniki, Greece.

[2] Biomic_AUTh, CIRI-AUTH Center for Interdisciplinary Research and Innovation Aristotle University of Thessaloniki, 57001, Thessaloniki, Greece.

[3] Second Department of Cardiology, General Hospital 'Hippokration', Aristotle University of Thessaloniki, Konstantinoupoleos 49, Thessaloniki 54642, Greece

[4] Athens Naval Hospital, Athens 11521, Greece

*Corresponding author: gkikae@auth.gr Laboratory of Forensic Medicine and Toxicology, School of Medicine, Aristotle University of Thessaloniki, 54124, Thessaloniki, Greece.

Contents:

Figure S1. PCA score plot for two studied groups and QC samples was constructed. Individual samples are demonstrated with grey color, whereas QC samples are depicted in green and clustered together (R2X=0.755, Q2=0.643)……………………………………………………2

Figure S2. Optimized model validation tests. A) Plot of permutation test results. Blue dots represent the performance of 100 models generated using the corresponding permuted response variables [SYNTAX score groups SS=0 (N=66), SS>0 (N=80)], while the blue square represents the performance of the reference model generated using the intact response variable. Model performance is assessed using the Matthews Correlation Coefficient (MCC) metric. The x-axis corresponds to the correlation of each permuted response variable with the intact one, using Spearman's correlation test. The performance of the test models is consistently significantly lower than that of the reference model, ruling out the possibility of random correlation of the predictor variable matrix with the response variable. b) Plot of model's learning curves. Learning curves are generated using a gradually increasing number of available samples (x-axis). The performance of the generated models is evaluated using the MCC metric. The results correspond to the average of 10 independent analyses using different randomization conditions. The validation curve increases gradually, steadily approaching the corresponding training curve, before reaching a plateau, indicating the absence of overfitting in the optimized model……3

Table S1. Summary of all identified lipids in the blood serum of patients with and without obstructive CAD (N=146). Information is provided regarding the lipid's species, molecular formula, monoisotopic mass, the adducts, retention time and the software tools used for identification…………………………………………………………………………………………………..4-21

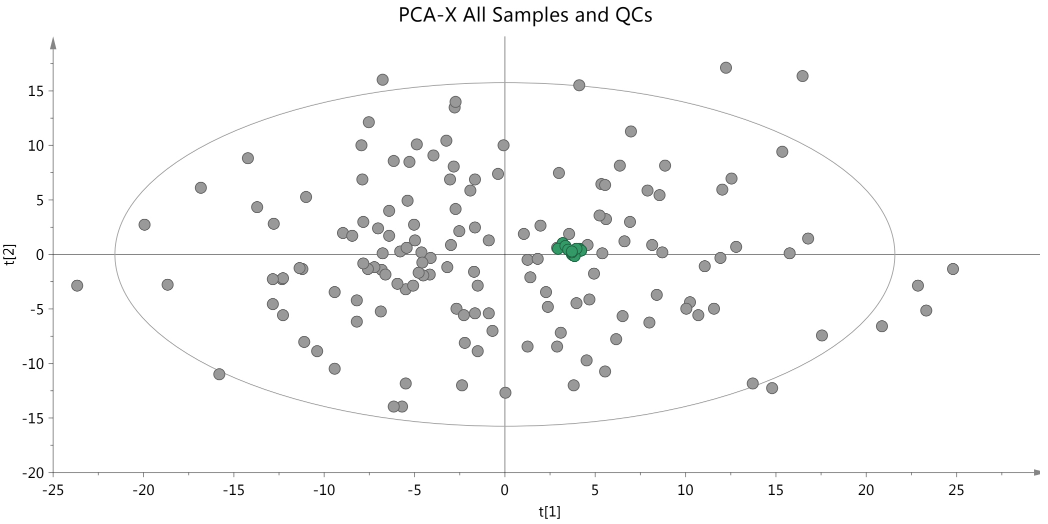

Figure S1. PCA score plot for two studied groups and QC samples was constructed. Individual samples are demonstrated with grey color, whereas QC samples are depicted in green and clustered together (R^2X=0.755, Q^2=0.643).

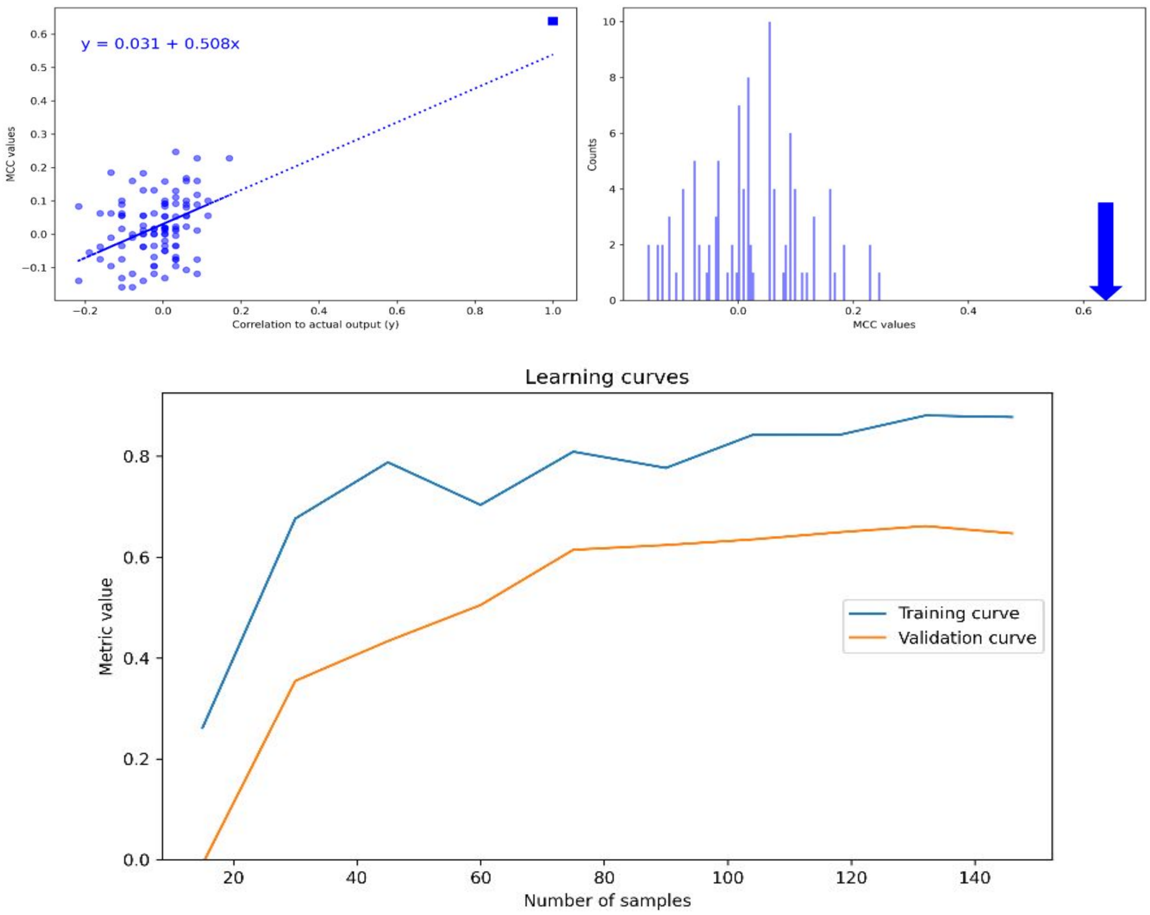

Figure S2. Optimized model validation tests. A) Plot of permutation test results. Blue dots represent the performance of 100 models generated using the corresponding permuted response variables [SYNTAX score groups SS=0 (N=66), SS>0 (N=80)], while the blue square represents the performance of the reference model generated using the intact response variable. Model performance is assessed using the Matthews Correlation Coefficient (MCC) metric. The x-axis corresponds to the correlation of each permuted response variable with the intact one, using Spearman's correlation test. The performance of the test models is consistently significantly lower than that of the reference model, ruling out the possibility of random correlation of the predictor variable matrix with the response variable. b) Plot of model's learning curves. Learning curves are generated using a gradually increasing number of available samples (x-axis). The performance of the generated models is evaluated using the MCC metric. The results correspond to the average of 10 independent analyses using different randomization conditions. The validation curve increases gradually, steadily approaching the corresponding training curve, before reaching a plateau, indicating the absence of overfitting in the optimized model.

Table S1. Summary of all identified lipids in the blood serum of patients with and without obstructive CAD (N=146). Information is provided regarding the lipid's species, molecular formula, monoisotopic mass, the adducts, retention time and the software tools used for identification.

Lipids Subclasses	Lipids Species	Molecular Species	Molecular Formula	Monoisoto-pic Mass	$[M+H]^+$	$[M-H_2O+H]^+$	$[M+NH_4]^+$	$[M+HCOO]^-$	$[M-H]^-$	RT	Lipostar	LipidHunter
**Acylcarnitines**												
CAR	CAR 10:0	CAR 10:0	C17H33NO4	315.2410	$[M+H]^+$					1.51	pos	
CAR	CAR 14:1	CAR 14:1	C21H39NO4	369.2879	$[M+H]^+$					3.05	pos	
CAR	CAR 16:0	CAR 16:0	C23H45NO4	399.3349	$[M+H]^+$					6.50	pos	
CAR	CAR 18:0	CAR 18:0	C25H49NO4	427.3662	$[M+H]^+$					9.08	pos	
CAR	CAR 18:1	CAR 18:1	C25H47NO4	425.3505	$[M+H]^+$					7.08	pos	
CAR	CAR 18:2	CAR 18:2	C25H45NO4	423.3349	$[M+H]^+$					5.44	pos	
**Ceramides**												
Cer	Cer(34:1;O2)	Cer(18:1;O2_16:0)	C34H67NO3	537.5121	$[M+H]^+$	$[M-H_2O+H]^+$		$[M+HCOO]^-$		18.8	pos/neg	
Cer	Cer(36:1;O2)	Cer(18:1;O2_18:0)	C36H71NO3	565.5434	$[M+H]^+$	$[M-H_2O+H]^+$		$[M+HCOO]^-$		19.9	pos/neg	
Cer	Cer(40:0;O2)	Cer(18:0;O2_22:0)	C40H81NO3	623.6216	$[M+H]^+$	$[M-H_2O+H]^+$		$[M+HCOO]^-$		22.1	pos/neg	pos
Cer	Cer(40:1;O2)	Cer(18:1;O2_22:0)	C40H79NO3	621.6060	$[M+H]^+$	$[M-H_2O+H]^+$		$[M+HCOO]^-$		21.8	pos/neg	pos
Cer	Cer(40:2;O2)	Cer(18:2;O2_22:0)	C40H77NO3	619.5903	$[M+H]^+$	$[M-H_2O+H]^+$		$[M+HCOO]^-$		21.1	pos/neg	
Cer	Cer(41:0;O2)	Cer(18:0;O2_23:0)	C41H83NO3	637.6373	$[M+H]^+$	$[M-H_2O+H]^+$		$[M+HCOO]^-$		22.5	pos/neg	
Cer	Cer(41:1;O2)	Cer(18:1;O2_23:0)	C41H81NO3	635.6216	$[M+H]^+$	$[M-H_2O+H]^+$		$[M+HCOO]^-$		22.2	pos/neg	pos
Cer	Cer(41:2;O2)	Cer(18:2;O2_23:0)	C41H79NO3	633.6060	$[M+H]^+$	$[M-H_2O+H]^+$		$[M+HCOO]^-$		21.6	pos/neg	pos
Cer	Cer(42:0;O2)	Cer(18:0;O2_24:0)	C42H85NO3	651.6529	$[M+H]^+$	$[M-H_2O+H]^+$		$[M+HCOO]^-$		22.8	pos/neg	
Cer	Cer(42:1;O2)	Cer(18:1;O2_24:0)	C42H83NO3	649.6373	$[M+H]^+$	$[M-H_2O+H]^+$		$[M+HCOO]^-$		22.6	pos/neg	
Cer	Cer(42:2;O2)	Cer(18:1;O2_24:1)	C42H81NO3	647.6216	$[M+H]^+$	$[M-H_2O+H]^+$		$[M+HCOO]^-$		21.7	pos/neg	pos
Cer	Cer(42:2;O2)	Cer(18:2;O2_24:0)	C42H81NO3	647.6216	$[M+H]^+$	$[M-H_2O+H]^+$		$[M+HCOO]^-$		22.0	pos/neg	pos
Cer	Cer(42:3;O2)	Cer(18:2;O2_24:1)	C42H79NO3	645.6060	$[M+H]^+$	$[M-H_2O+H]^+$		$[M+HCOO]^-$		21.0	pos/neg	
Cer	Cer(43:1;O2)	Cer(18:1;O2_25:0)	C43H85NO3	663.6529	$[M+H]^+$	$[M-H_2O+H]^+$		$[M+HCOO]^-$		22.9	pos/neg	pos
Cer	Cer(43:2;O2)	Cer(18:2;O2_25:0)	C43H83NO3	661.6373	$[M+H]^+$	$[M-H_2O+H]^+$		$[M+HCOO]^-$		22.0	pos/neg	
Cer	Cer(44:1;O2)	Cer(18:1;O2_26:0)	C44H87NO3	677.6686	$[M+H]^+$	$[M-H_2O+H]^+$		$[M+HCOO]^-$		23.3	pos/neg	pos
**Hex-(n)-Cer**												

Lipids Subclasses	Lipids Species	Molecular Species	Molecular Formula	Monoisoto -pic Mass	[M+H]$^+$	[M-H$_2$O+H]$^+$	[M+NH$_4$]$^+$	[M+HCOO]$^-$	[M-H]$^-$	RT	Lipostar	LipidHunter
Hex1Cer	HexCer(34:1;O2)	HexCer(18:1;O2_16:0)	C40H77NO8	699.5649	[M+H]$^+$	[M-H$_2$O+H]$^+$		[M+HCOO]$^-$		17.8	neg	pos
Hex1Cer	HexCer(40:1;O2)	HexCer(18:1;O2_22:0)	C46H89NO8	783.6588	[M+H]$^+$	[M-H$_2$O+H]$^+$		[M+HCOO]$^-$		21.0	neg	
Hex1Cer	HexCer(40:2;O2)	HexCer(16:2;O2_24:0)	C46H87NO8	781.6588	[M+H]$^+$	[M-H$_2$O+H]$^+$		[M+HCOO]$^-$		20.3	neg	
Hex1Cer	HexCer(41:1;O2)	HexCer(18:1;O2_23:0)	C47H91NO8	797.6744	[M+H]$^+$	[M-H$_2$O+H]$^+$		[M+HCOO]$^-$		21.5	neg	
Hex1Cer	HexCer(42:1;O2)	HexCer(18:1;O2_24:0)	C48H93NO8	811.6901	[M+H]$^+$	[M-H$_2$O+H]$^+$		[M+HCOO]$^-$		21.9	neg	
Hex1Cer	HexCer(42:2;O2)	HexCer(18:1;O2_24:1)	C48H91NO8	809.6744	[M+H]$^+$	[M-H$_2$O+H]$^+$		[M+HCOO]$^-$		20.9	neg	
Hex1Cer	HexCer(34:1;O3)	HexCer(18:1;O2_16:0;O)	C40H77NO8	715.5598	[M+H]$^+$	[M-H$_2$O+H]$^+$		[M+HCOO]$^-$		17.4	neg	
Hex1Cer	HexCer(42:1;O3)	HexCer(18:1;O2_24:0;O)	C48H93NO9	827.6850	[M+H]$^+$	[M-H$_2$O+H]$^+$		[M+HCOO]$^-$		21.6	neg	
Hex2Cer	Hex2Cer(34:1;O2)	Hex2Cer(18:1;O2_16:0)	C46H87NO13	861.6177	[M+H]$^+$	[M-H$_2$O+H]$^+$		[M+HCOO]$^-$		17.4	neg	pos
**Sphingomyelins**												
SM	SM(30:1;O2)	SM(30:1;O2)	C35H71N2O6P	646.5049	[M+H]$^+$	[M-H$_2$O+H]$^+$				14.3		pos
SM	SM(32:0;O2)	SM(32:0;O2)	C37H77N2O6P	676.5362	[M+H]$^+$	[M-H$_2$O+H]$^+$		[M+HCOO]$^-$		16.4	neg	pos
SM	SM(32:1;O2)	SM(32:1;O2)	C37H75N2O6P	674.5362	[M+H]$^+$	[M-H$_2$O+H]$^+$		[M+HCOO]$^-$		15.9	neg	pos
SM	SM(32:2;O2)	SM(32:2;O2)	C37H73N2O6P	672.5206	[M+H]$^+$	[M-H$_2$O+H]$^+$		[M+HCOO]$^-$		14.6	neg	pos
SM	SM(33:1;O2)	SM(33:1;O2)	C38H77N2O6P	688.5519	[M+H]$^+$	[M-H$_2$O+H]$^+$		[M+HCOO]$^-$		16.6	neg	pos
SM	SM(34:0;O2)	SM(34:0;O2)	C39H81N2O6P	704.5832	[M+H]$^+$	[M-H$_2$O+H]$^+$		[M+HCOO]$^-$		17.8	neg	pos
SM	SM(34:1;O2)	SM(34:1;O2)	C39H79N2O6P	702.5675	[M+H]$^+$	[M-H$_2$O+H]$^+$		[M+HCOO]$^-$		17.3	neg	pos
SM	SM(34:2;O2)	SM(34:2;O2)	C39H77N2O6P	700.5519	[M+H]$^+$	[M-H$_2$O+H]$^+$		[M+HCOO]$^-$		16.2	neg	pos
SM	SM(35:1;O2)	SM(35:1;O2)	C40H81N2O6P	716.5832	[M+H]$^+$	[M-H$_2$O+H]$^+$		[M+HCOO]$^-$		17.9	neg	pos
SM	SM(36:0;O2)	SM(36:0;O2)	C41H85N2O6P	732.6145	[M+H]$^+$	[M-H$_2$O+H]$^+$		[M+HCOO]$^-$		19.0	neg	pos
SM	SM(36:1;O2)	SM(36:1;O2)	C41H83N2O6P	730.5988	[M+H]$^+$	[M-H$_2$O+H]$^+$		[M+HCOO]$^-$		18.6	neg	pos
SM	SM(36:2;O2)	SM(36:2;O2)	C41H81N2O6P	728.5832	[M+H]$^+$	[M-H$_2$O+H]$^+$		[M+HCOO]$^-$		17.6	neg	pos
SM	SM(36:3;O2)	SM(36:3;O2)	C41H79N2O6P	726.5675	[M+H]$^+$	[M-H$_2$O+H]$^+$		[M+HCOO]$^-$		16.6	neg	
SM	SM(37:1;O2)	SM(37:1;O2)	C42H85N2O6P	744.6145	[M+H]$^+$	[M-H$_2$O+H]$^+$		[M+HCOO]$^-$		19.2	neg	pos
SM	SM(37:2;O2)	SM(37:2;O2)	C42H83N2O6P	742.5988	[M+H]$^+$	[M-H$_2$O+H]$^+$				18.3		pos
SM	SM(38:0;O2)	SM(38:0;O2)	C43H89N2O6P	760.6458	[M+H]$^+$	[M-H$_2$O+H]$^+$		[M+HCOO]$^-$		20.1	neg	
SM	SM(38:1;O2)	SM(38:1;O2)	C43H87N2O6P	758.6301	[M+H]$^+$	[M-H$_2$O+H]$^+$		[M+HCOO]$^-$		19.7	neg	
SM	SM(38:2;O2)	SM(38:2;O2)	C43H85N2O6P	756.6145	[M+H]$^+$	[M-H$_2$O+H]$^+$		[M+HCOO]$^-$		18.9	neg	pos
SM	SM(39:1;O2)	SM(39:1;O2)	C44H89N2O6P	772.6458	[M+H]$^+$	[M-H$_2$O+H]$^+$		[M+HCOO]$^-$		20.3	neg	
SM	SM(39:2;O2)	SM(39:2;O2)	C44H87N2O6P	770.6301	[M+H]$^+$	[M-H$_2$O+H]$^+$		[M+HCOO]$^-$		19.4	neg	pos

Lipids Subclasses	Lipids Species	Molecular Species	Molecular Formula	Monoisoto-pic Mass	$[M+H]^+$	$[M-H_2O+H]^+$	$[M+NH_4]^+$	$[M+HCOO]^-$	$[M-H]^-$	RT	Lipostar	LipidHunter
SM	SM(40:1;O2)	SM(40:1;O2)	C45H91N2O6P	786.6614	$[M+H]^+$	$[M-H_2O+H]^+$		$[M+HCOO]^-$		20.7	neg	
SM	SM(40:2;O2)	SM(40:2;O2)	C45H89N2O6P	784.6458	$[M+H]^+$	$[M-H_2O+H]^+$		$[M+HCOO]^-$		20.0	neg	
SM	SM(40:3;O2)	SM(40:3;O2)	C45H87N2O6P	782.6301	$[M+H]^+$	$[M-H_2O+H]^+$		$[M+HCOO]^-$		18.9	neg	
SM	SM(41:1;O2)	SM(41:1;O2)	C46H93N2O6P	800.6771	$[M+H]^+$	$[M-H_2O+H]^+$		$[M+HCOO]^-$		21.2	neg	
SM	SM(41:2;O2)	SM(41:2;O2)	C46H91N2O6P	798.6614	$[M+H]^+$	$[M-H_2O+H]^+$		$[M+HCOO]^-$		20.5	neg	
SM	SM(42:1;O2)	SM(42:1;O2)	C47H95N2O6P	814.6927	$[M+H]^+$	$[M-H_2O+H]^+$		$[M+HCOO]^-$		21.6	neg	
SM	SM(42:2;O2)	SM(42:2;O2)	C47H93N2O6P	812.6771	$[M+H]^+$	$[M-H_2O+H]^+$		$[M+HCOO]^-$		20.6	neg	
SM	SM(42:3;O2)	SM(42:3;O2)	C47H91N2O6P	810.6614	$[M+H]^+$	$[M-H_2O+H]^+$		$[M+HCOO]^-$		19.9	neg	
SM	SM(43:1;O2)	SM(43:1;O2)	C48H97N2O6P	828.7084	$[M+H]^+$	$[M-H_2O+H]^+$		$[M+HCOO]^-$		21.8	neg	
SM	SM(43:2;O2)	SM(43:2;O2)	C48H95N2O6P	826.6927	$[M+H]^+$	$[M-H_2O+H]^+$		$[M+HCOO]^-$		20.9	neg	
SM	SM(44:2;O2)	SM(44:2;O2)	C49H97N2O6P	840.7084	$[M+H]^+$	$[M-H_2O+H]^+$				20.2	neg	
**Phospholipids**												
**LPC**												
LPC	LPC(14:0)	LPC(14:0)	C22H46NO7P	467.3011	$[M+H]^+$			$[M+HCOO]^-$		3.95	pos	neg
LPC	LPC(15:0)	LPC(15:0)	C23H48NO7P	481.3168	$[M+H]^+$					5.24	pos	
LPC	LPC(16:0)	LPC(16:0)	C24H50NO7P	495.3324	$[M+H]^+$			$[M+HCOO]^-$		6.71	pos/neg	neg
LPC	LPC(16:1)	LPC(16:1)	C24H48NO7P	493.3168	$[M+H]^+$					4.62	pos	
LPC	LPC(17:0)	LPC(17:0)	C25H52NO7P	509.3481	$[M+H]^+$			$[M+HCOO]^-$		8.22	pos	neg
LPC	LPC(18:0)	LPC(18:0)	C26H54NO7P	523.3637	$[M+H]^+$			$[M+HCOO]^-$		9.67	pos/neg	neg
LPC	LPC(18:1)	LPC(18:1)	C26H52NO7P	521.3481	$[M+H]^+$			$[M+HCOO]^-$		7.39	pos/neg	neg
LPC	LPC(18:2)	LPC(18:2)	C26H50NO7P	519.3324	$[M+H]^+$			$[M+HCOO]^-$		5.51	pos/neg	neg
LPC	LPC(20:0)	LPC(20:0)	C28H58NO7P	551.3950	$[M+H]^+$					12.3	pos	
LPC	LPC(20:1)	LPC(20:1)	C28H56NO7P	549.3794	$[M+H]^+$					10.0	pos	
LPC	LPC(20:2)	LPC(20:2)	C28H54NO7P	547.3637	$[M+H]^+$					8.12	pos	
LPC	LPC(20:3)	LPC(20:3)	C28H52NO7P	545.3481	$[M+H]^+$			$[M+HCOO]^-$		6.52	pos/neg	neg
LPC	LPC(20:4)	LPC(20:4)	C28H50NO7P	543.3324	$[M+H]^+$			$[M+HCOO]^-$		5.48	pos/neg	neg
LPC	LPC(20:5)	LPC(20:5)	C28H48NO7P	541.3168	$[M+H]^+$					4.12	pos	
LPC	LPC(22:5)	LPC(22:5)	C30H52NO7P	569.3481	$[M+H]^+$					6.79	pos	
LPC	LPC(22:6)	LPC(22:6)	C30H50NO7P	567.3324	$[M+H]^+$			$[M+HCOO]^-$		5.32	pos/neg	neg
LPC	LPC(24:0)	LPC(24:0)	C32H66NO7P	607.4576	$[M+H]^+$					16.2	pos	
LPC	LPC(O-16:0)	LPC(O-16:0)	C24H52NO6P	481.3532	$[M+H]^+$					7.78	pos	

Lipids Subclasses	Lipids Species	Molecular Species	Molecular Formula	Monoisoto-pic Mass	$[M+H]^+$	$[M-H_2O+H]^+$	$[M+NH_4]^+$	$[M+HCOO]^-$	$[M-H]^-$	RT	Lipostar	LipidHunter
LPC	LPC(P-16:0)	LPC(P-16:0)	C24H50NO6P	479.3375	$[M+H]^+$					7.71	pos	
LPC	LPC(O-18:1)	LPC(O-18:1)	C26H54NO6P	507.3688	$[M+H]^+$					8.38	pos	
**LPE**												
LPE	LPE(16:0)	LPE(16:0)	C21H44NO7P	453.2855	$[M+H]^+$				[M-H]-	6.92		neg
LPE	LPE(18:0)	LPE(18:0)	C23H48NO7P	481.3168	$[M+H]^+$				[M-H]-	9.93	pos/neg	neg
LPE	LPE(18:1)	LPE(18:1)	C23H46NO7P	479.3011	$[M+H]^+$				[M-H]-	7.62	pos/neg	
LPE	LPE(18:2)	LPE(18:2)	C23H44NO7P	477.2855	$[M+H]^+$				[M-H]-	5.67	pos/neg	neg
LPE	LPE(20:4)	LPE(20:4)	C25H44NO7P	501.2855	$[M+H]^+$				[M-H]-	5.64	pos/neg	neg
LPE	LPE(22:6)	LPE(22:6)	C27H44NO7P	525.2855	$[M+H]^+$				[M-H]-	5.47	pos/neg	
**PC**												
PC	PC(28:0)		C36H72NO8P	677.4995	$[M+H]^+$					16.1	pos	
PC	PC(30:0)	PC(14:0_16:0)	C38H76NO8P	705.5308	$[M+H]^+$			$[M+HCOO]^-$		17.4	pos/neg	
PC	PC(30:1)		C38H74NO8P	703.5152	$[M+H]^+$					16.4	pos	
PC	PC(31:0)	PC(15:0_16:0)	C39H78NO8P	719.5465	$[M+H]^+$			$[M+HCOO]^-$		18.0	pos/neg	
PC	PC(32:0)	PC(16:0_16:0)	C40H80NO8P	733.5621	$[M+H]^+$			$[M+HCOO]^-$		18.6	pos/neg	
PC	PC(32:1)	PC(16:0_16:1)	C40H78NO8P	731.5465	$[M+H]^+$			$[M+HCOO]^-$		17.7	pos/neg	
PC	PC(32:2)	PC(14:0_18:2)	C40H76NO8P	729.5308	$[M+H]^+$			$[M+HCOO]^-$		16.7	pos	neg
PC	PC(33:0)	PC(16:0_17:0)	C41H82NO8P	747.5778	$[M+H]^+$			$[M+HCOO]^-$		19.0	pos/neg	neg
PC	PC(33:1)	PC(15:0_18:1)	C41H80NO8P	745.5621	$[M+H]^+$			$[M+HCOO]^-$		18.2	pos/neg	
PC	PC(33:2)	PC(15:0_18:2)	C41H78NO8P	743.5465	$[M+H]^+$			$[M+HCOO]^-$		17.4	pos/neg	neg
PC	PC(34:0)	PC(16:0_18:0)	C42H84NO8P	761.5934	$[M+H]^+$			$[M+HCOO]^-$		19.7	pos/neg	
PC	PC(34:1)	PC(16:0_18:1)	C42H82NO8P	759.5778	$[M+H]^+$			$[M+HCOO]^-$		18.8	pos/neg	neg
PC	PC(34:2)	PC(16:0_18:2)	C42H80NO8P	757.5621	$[M+H]^+$			$[M+HCOO]^-$		18.0		neg
PC	PC(34:2)	PC(16:1_18:1)	C42H80NO8P	757.5621	$[M+H]^+$			$[M+HCOO]^-$		17.8	pos/neg	neg
PC	PC(34:3)	PC(16:1_18:2)	C42H78NO8P	755.5465	$[M+H]^+$			$[M+HCOO]^-$		16.9		neg
PC	PC(34:3)	PC(16:0_18:3)	C42H78NO8P	755.5465	$[M+H]^+$			$[M+HCOO]^-$		17.4	pos/neg	neg
PC	PC(34:4)	PC(14:0_20:4)	C42H76NO8P	753.5308	$[M+H]^+$			$[M+HCOO]^-$		16.6	pos/neg	neg
PC	PC(34:5)		C42H74NO8P	751.5152	$[M+H]^+$					15.8	pos	
PC	PC(35:1)	PC(17:0_18:1)	C43H84NO8P	773.5934	$[M+H]^+$			$[M+HCOO]^-$		19.3	pos/neg	neg
PC	PC(35:2)	PC(17:1_18:1)	C43H82NO8P	771.5778	$[M+H]^+$			$[M+HCOO]^-$		18.4	pos/neg	
PC	PC(35:2)	PC(17:0_18:2)	C43H82NO8P	771.5778	$[M+H]^+$			$[M+HCOO]^-$		18.6		neg

Lipids Subclasses	Lipids Species	Molecular Species	Molecular Formula	Monoisoto-pic Mass	$[M+H]^+$	$[M-H_2O+H]^+$	$[M+NH_4]^+$	$[M+HCOO]^-$	$[M-H]^-$	RT	Lipostar	LipidHunter
PC	PC(35:3)	PC(17:1_18:2)	C43H80NO8P	769.5621	$[M+H]^+$			$[M+HCOO]^-$		17.5		neg
PC	PC(35:3)	PC(15:0_20:3)	C43H80NO8P	769.5621	$[M+H]^+$			$[M+HCOO]^-$		17.7	pos/neg	neg
PC	PC(35:4)	PC(15:0_20:4)	C43H78NO8P	767.5465	$[M+H]^+$			$[M+HCOO]^-$		17.3	pos/neg	neg
PC	PC(36:1)	PC(18:0_18:1)	C44H86NO8P	787.6091	$[M+H]^+$			$[M+HCOO]^-$		19.9	pos/neg	neg
PC	PC(36:2)	PC(18:1_18:1)	C44H84NO8P	785.5934	$[M+H]^+$			$[M+HCOO]^-$		18.9		neg
PC	PC(36:2)	PC(18:0_18:2)	C44H84NO8P	785.5934	$[M+H]^+$			$[M+HCOO]^-$		19.1	pos/neg	neg
PC	PC(36:3)	PC(16:0_20:3)	C44H82NO8P	783.5778	$[M+H]^+$			$[M+HCOO]^-$		18.3		neg
PC	PC(36:3)	PC(18:1_18:2)	C44H82NO8P	783.5778	$[M+H]^+$			$[M+HCOO]^-$		18.1	pos/neg	neg
PC	PC(36:4)	PC(16:0_20:4)	C44H80NO8P	781.5621	$[M+H]^+$			$[M+HCOO]^-$		17.9	pos/neg	neg
PC	PC(36:5)	PC(16:0_20:5)	C44H78NO8P	779.5465	$[M+H]^+$			$[M+HCOO]^-$		17.2	pos/neg	neg
PC	PC(36:6)	PC(14:0_22:6)	C44H76NO8P	777.5308	$[M+H]^+$			$[M+HCOO]^-$		16.4	pos/neg	neg
PC	PC(37:1)		C45H88NO8P	801.6247	$[M+H]^+$					20.3	pos	
PC	PC(37:2)	PC(18:2_19:0)	C45H86NO8P	799.6091	$[M+H]^+$			$[M+HCOO]^-$		19.7	pos/neg	neg
PC	PC(37:3)	PC(17:0_20:3)	C45H84NO8P	797.5934	$[M+H]^+$			$[M+HCOO]^-$		18.9	pos	neg
PC	PC(37:4)	PC(17:0_20:4)	C45H82NO8P	795.5778	$[M+H]^+$			$[M+HCOO]^-$		18.3	pos/neg	neg
PC	PC(37:5)		C45H80NO8P	793.5621	$[M+H]^+$					17.4	pos	
PC	PC(37:6)	PC(15:0_22:6)	C45H78NO8P	791.5465	$[M+H]^+$			$[M+HCOO]^-$		17.0	pos/neg	neg
PC	PC(38:1)	PC(18:0_20:1)	C46H90NO8P	815.6404	$[M+H]^+$			$[M+HCOO]^-$		20.7	pos/neg	
PC	PC(38:2)	PC(18:0_20:2)	C46H88NO8P	813.6247	$[M+H]^+$			$[M+HCOO]^-$		20.0	pos/neg	
PC	PC(38:3)	PC(18:0_20:3)	C46H86NO8P	811.6091	$[M+H]^+$			$[M+HCOO]^-$		19.4	pos/neg	neg
PC	PC(38:4)	PC(18:1_20:3)	C46H84NO8P	809.5934	$[M+H]^+$			$[M+HCOO]^-$		18.6		neg
PC	PC(38:4)	PC(18:0_20:4)	C46H84NO8P	809.5934	$[M+H]^+$			$[M+HCOO]^-$		19.0	pos/neg	neg
PC	PC(38:5)	PC(18:0_20:5)	C46H82NO8P	807.5778	$[M+H]^+$			$[M+HCOO]^-$		18.4		neg
PC	PC(38:5)	PC(18:1_20:4)	C46H82NO8P	807.5778	$[M+H]^+$			$[M+HCOO]^-$		18.1		neg
PC	PC(38:5)	PC(16:0_22:5)	C46H82NO8P	807.5778	$[M+H]^+$			$[M+HCOO]^-$		18.4	pos/neg	
PC	PC(38:6)	PC(18:2_20:4)	C46H80NO8P	805.5621	$[M+H]^+$			$[M+HCOO]^-$		17.2		neg
PC	PC(38:6)	PC(16:0_22:6)	C46H80NO8P	805.5621	$[M+H]^+$			$[M+HCOO]^-$		17.7	pos/neg	neg
PC	PC(38:7)	PC(16:1_22:6)	C46H78NO8P	803.5465	$[M+H]^+$			$[M+HCOO]^-$		16.6	neg	
PC	PC(39:6)		C47H82NO8P	819.5778	$[M+H]^+$					18.3	pos	
PC	PC(39:6)	PC(17:0_22:6)	C47H82NO8P	819.5778	$[M+H]^+$			$[M+HCOO]^-$		18.1		neg
PC	PC(40:3)		C48H90NO8P	839.6404	$[M+H]^+$					20.4	pos	

Lipids Subclasses	Lipids Species	Molecular Species	Molecular Formula	Monoisoto -pic Mass	$[M+H]^+$	$[M-H_2O+H]^+$	$[M+NH_4]^+$	$[M+HCOO]^-$	$[M-H]^-$	RT	Lipostar	LipidHunter
PC	PC(40:4)	PC(18:0_22:4)	C48H88NO8P	837.6247	$[M+H]^+$			$[M+HCOO]^-$		19.8	pos/neg	neg
PC	PC(40:5)	PC(18:0_22:5)	C48H86NO8P	835.6091	$[M+H]^+$			$[M+HCOO]^-$		19.5	pos/neg	neg
PC	PC(40:6)	PC(18:0_22:6)	C48H84NO8P	833.5934	$[M+H]^+$			$[M+HCOO]^-$		18.8	pos/neg	neg
PC	PC(40:7)	PC(18:1_22:6)	C48H82NO8P	831.5778	$[M+H]^+$			$[M+HCOO]^-$		17.8	pos/neg	neg
PC	PC(40:8)	PC(18:2_22:6)	C48H80NO8P	829.5621	$[M+H]^+$			$[M+HCOO]^-$		17.0	pos/neg	
PC	PC(42:10)		C50H80NO8P	853.5621	$[M+H]^+$					16.7	pos	
PC	PC(42:2)		C50H96NO8P	869.6873	$[M+H]^+$					22.0	pos	
PC	PC(42:4)		C50H92NO8P	865.6560	$[M+H]^+$					20.5	pos	
PC	PC(42:6)		C50H88NO8P	861.6247	$[M+H]^+$					19.5	pos	
PC	PC(42:7)		C50H86NO8P	859.6091	$[M+H]^+$					18.9	pos	
PC	PC(42:8)		C50H84NO8P	857.5934	$[M+H]^+$					18.0	pos	
PC	PC(O-30:0)		C38H78NO7P	691.5515	$[M+H]^+$					18.2	pos	
PC	PC(O-32:0)		C40H82NO7P	719.5828	$[M+H]^+$					19.3	pos	
PC	PC(O-34:0)	PC(O-18:0_16:0)	C42H86NO7P	747.6141	$[M+H]^+$			$[M+HCOO]^-$		20.4	pos	neg
PC	PC(O-34:1)	PC(O-16:0_18:1)	C42H84NO7P	745.5985	$[M+H]^+$			$[M+HCOO]^-$		19.5	pos/neg	neg
PC	PC(O-34:2)	PC(O-16:0_18:2)	C42H82NO7P	743.5828	$[M+H]^+$			$[M+HCOO]^-$		18.7		neg
PC	PC(O-34:3)	PC(O-16:1_18:2)	C42H80NO7P	741.5828	$[M+H]^+$			$[M+HCOO]^-$		18.5	pos/neg	neg
PC	PC(O-36:2)	PC(O-18:0_18:2)	C44H86NO7P	771.6141	$[M+H]^+$			$[M+HCOO]^-$		19.8	pos/neg	neg
PC	PC(O-36:4)	PC(O-16:0_20:4)	C44H82NO7P	767.5828	$[M+H]^+$			$[M+HCOO]^-$		18.6	pos/neg	neg
PC	PC(O-37:4)		C45H84NO7P	781.5985	$[M+H]^+$					19.2	pos	
PC	PC(O-38:3)	PC(O-18:0_20:3)	C46H88NO7P	797.6298	$[M+H]^+$			$[M+HCOO]^-$		20.1	pos/neg	neg
PC	PC(O-38:4)	PC(O-16:0_22:4)	C46H86NO7P	795.6141	$[M+H]^+$			$[M+HCOO]^-$		19.4		neg
PC	PC(O-38:4)	PC(O-18:0_20:4)	C46H86NO7P	795.6141	$[M+H]^+$			$[M+HCOO]^-$		19.7	pos/neg	neg
PC	PC(O-38:5)	PC(O-18:1_20:4)	C46H84NO7P	793.5985	$[M+H]^+$			$[M+HCOO]^-$		18.7	pos/neg	neg
PC	PC(O-38:6)	PC(O-16:0_22:6)	C46H82NO7P	791.5828	$[M+H]^+$			$[M+HCOO]^-$		18.4	pos/neg	neg
PC	PC(O-40:4)	PC(O-20:0_20:4)	C48H90NO7P	823.6454	$[M+H]^+$			$[M+HCOO]^-$		20.7	pos/neg	
PC	PC(O-40:5)	PC(O-20:1_20:4)	C48H88NO7P	821.6298	$[M+H]^+$			$[M+HCOO]^-$		19.7	pos/neg	
PC	PC(O-42:2)		C50H98NO7P	855.7080	$[M+H]^+$					22.1	pos	
PC	PC(O-42:4)		C50H94NO7P	851.6767	$[M+H]^+$					21.6	pos	
PC	PC(O-42:6)		C50H90NO7P	847.6454	$[M+H]^+$					19.9	pos	
PC	PC(O-44:4)		C52H98NO7P	879.7080	$[M+H]^+$					22.4	pos	

Lipids Subclasses	Lipids Species	Molecular Species	Molecular Formula	Monoisoto-pic Mass	$[M+H]^+$	$[M-H_2O+H]^+$	$[M+NH_4]^+$	$[M+HCOO]^-$	$[M-H]^-$	RT	Lipostar	LipidHunter
PC	PC(O-44:5)		C52H96NO7P	877.6924	$[M+H]^+$					21.5	pos	
PC	PC(P-30:0)		C38H76NO7P	689.5359	$[M+H]^+$					18.0	pos	
PC	PC(P-32:0)	PC(P-16:0_16:0)	C40H80NO7P	717.5672	$[M+H]^+$			$[M+HCOO]^-$		19.2	pos/neg	neg
PC	PC(P-34:1)	PC(P-16:0_18:1)	C42H82NO7P	743.5828	$[M+H]^+$			$[M+HCOO]^-$		19.3	pos/neg	neg
PC	PC(P-34:2)	PC(P-16:0_18:2)	C42H80NO7P	741.5672	$[M+H]^+$			$[M+HCOO]^-$		18.5		neg
PC	PC(P-34:4)		C42H76NO7P	737.5359	$[M+H]^+$					17.4	pos	
PC	PC(P-35:2)	PC(P-17:0_18:2)	C43H82NO7P	755.5828	$[M+H]^+$					19.1	pos	
PC	PC(P-36:1)	PC(P-18:0_18:1)	C44H86NO7P	771.6141	$[M+H]^+$			$[M+HCOO]^-$		19.6		neg
PC	PC(P-36:4)	PC(P-16:0_20:4)	C44H80NO7P	765.5672	$[M+H]^+$			$[M+HCOO]^-$		18.4	pos/neg	neg
PC	PC(P-38:4)	PC(P-16:0_22:4)	C46H84NO7P	793.5985	$[M+H]^+$			$[M+HCOO]^-$		19.2		neg
PC	PC(P-38:6)	PC(P-16:0_22:6)	C46H80NO7P	789.5672	$[M+H]^+$			$[M+HCOO]^-$		18.2	pos/neg	neg
PC	PC(P-42:4)		C50H92NO7P	849.6611	$[M+H]^+$					20.6	pos	
PC	PC(P-42:6)		C50H88NO7P	845.6298	$[M+H]^+$					19.5	pos	
					**PE**							
PE	PE(34:1)	PE(16:0_18:1)	C39H76NO8P	717.5308	$[M+H]^+$				$[M-H]^-$	19.1		neg
PE	PE(34:2)	PE(16:0_18:2)	C39H74NO8P	715.5152	$[M+H]^+$				$[M-H]^-$	18.3	pos/neg	neg
PE	PE(36:1)	PE(18:0_18:1)	C41H80NO8P	745.5621	$[M+H]^+$				$[M-H]^-$	20.1	pos/neg	neg
PE	PE(36:2)	PE(18:1_18:1)	C41H78NO8P	743.5465	$[M+H]^+$				$[M-H]^-$	19.2	pos/neg	neg
PE	PE(36:2)	PE(18:0_18:2)	C41H78NO8P	743.5465	$[M+H]^+$				$[M-H]^-$	19.4	neg	neg
PE	PE(36:3)	PE(16:0_20:3)	C41H76NO8P	741.5308	$[M+H]^+$				$[M-H]^-$	18.6	pos/neg	neg
PE	PE(36:3)	PE(18:1_18:2)	C41H76NO8P	741.5308	$[M+H]^+$				$[M-H]^-$	18.4		neg
PE	PE(36:4)	PE(16:0_20:4)	C41H74NO8P	739.5152	$[M+H]^+$				$[M-H]^-$	18.2	pos/neg	neg
PE	PE(38:3)	PE(18:0_20:3)	C43H80NO8P	769.5621	$[M+H]^+$				$[M-H]^-$	19.7	neg	neg
PE	PE(38:5)	PE(18:1_20:4)	C43H76NO8P	765.5308	$[M+H]^+$				$[M-H]^-$	18.3	pos/neg	neg
PE	PE(38:5)	PE(18:0_20:5)	C43H76NO8P	765.5308	$[M+H]^+$				$[M-H]^-$	18.7		neg
PE	PE(38:6)	PE(16:0_22:6)	C43H74NO8P	763.5152	$[M+H]^+$				$[M-H]^-$	18.0	pos/neg	neg
PE	PE(40:4)	PE(18:0_22:4)	C45H82NO8P	795.5778	$[M+H]^+$				$[M-H]^-$	20.0		neg
PE	PE(40:6)	PE(18:0_22:6)	C45H78NO8P	791.5465	$[M+H]^+$				$[M-H]^-$	19.1	pos/neg	neg
PE	PE(40:7)	PE(18:1_22:6)	C45H76NO8P	789.5308	$[M+H]^+$				$[M-H]^-$	18.1		neg
PE	PE(O-36:4)	PE(O-16:0_20:4)	C41H76NO7P	725.5359	$[M+H]^+$				$[M-H]^-$	18.9		neg
PE	PE(P-34:1)	PE(P-16:0_18:1)	C39H76NO7P	701.5359	$[M+H]^+$				$[M-H]^-$	19.6	pos/neg	neg

Lipids Subclasses	Lipids Species	Molecular Species	Molecular Formula	Monoisoto -pic Mass	$[M+H]^+$	$[M-H_2O+H]^+$	$[M+NH_4]^+$	$[M+HCOO]^-$	$[M-H]^-$	RT	Lipostar	LipidHunter
PE	PE(P-34:2)	PE(P-16:0_18:2)	C39H74NO7P	699.5202	$[M+H]^+$				$[M-H]^-$	18.8	pos/neg	neg
PE	PE(P-36:1)	PE(P-18:0_18:1)	C41H80NO7P	729.5672	$[M+H]^+$				$[M-H]^-$	20.6	pos	neg
PE	PE(P-36:2)	PE(P-18:0_18:2)	C41H78NO7P	727.5515	$[M+H]^+$				$[M-H]^-$	19.9	pos	neg
PE	PE(P-36:3)	PE(P-16:0_20:3)	C41H76NO7P	725.5359	$[M+H]^+$				$[M-H]^-$	19.2	pos/neg	
PE	PE(P-36:4)	PE(P-16:0_20:4)	C41H74NO7P	723.5202	$[M+H]^+$				$[M-H]^-$	18.7	pos/neg	neg
PE	PE(P-38:3)	PE(P-18:0_20:3)	C43H80NO7P	753.5672	$[M+H]^+$				$[M-H]^-$	20.2	pos	neg
PE	PE(P-38:4)	PE(P-18:0_20:4)	C43H78NO7P	751.5515	$[M+H]^+$				$[M-H]^-$	19.8	pos	neg
PE	PE(P-38:4)	PE(P-16:0_22:4)	C43H78NO7P	751.5515	$[M+H]^+$				$[M-H]^-$	19.5	pos	neg
PE	PE(P-38:5)	PE(P-18:0_20:5)	C43H76NO7P	749.5359	$[M+H]^+$				$[M-H]^-$	19.2		neg
PE	PE(P-38:5)	PE(P-16:0_22:5)	C43H76NO7P	749.5359	$[M+H]^+$				$[M-H]^-$	18.9		neg
PE	PE(P-38:6)	PE(P-16:0_22:6)	C43H74NO7P	747.5202	$[M+H]^+$				$[M-H]^-$	18.5	pos/neg	neg
PE	PE(P-40:4)	PE(P-20:0_20:4)	C45H82NO7P	779.5828	$[M+H]^+$				$[M-H]^-$	20.8	pos	neg
PE	PE(P-40:6)	PE(P-18:0_22:6)	C45H78NO7P	775.5515	$[M+H]^+$				$[M-H]^-$	19.6	pos/neg	neg
PE	PE(P-40:7)	PE(P-18:1_22:6)	C45H76NO7P	773.5359	$[M+H]^+$				$[M-H]^-$	18.6	pos/neg	
						**PI**						
PI	PI(32:1)	PI(16:0_16:1)	C41H77O13P	808.5101					$[M-H]^-$	16.8	neg	neg
PI	PI(34:1)	PI(16:0_18:1)	C43H81O13P	836.5414					$[M-H]^-$	18.0	neg	
PI	PI(34:2)	PI(16:1_18:1)	C43H79O13P	834.5258	$[M+H]^+$				$[M-H]^-$	17.1	pos/neg	neg
PI	PI(36:1)	PI(18:0_18:1)	C45H85O13P	864.5727					$[M-H]^-$	19.0	neg	neg
PI	PI(36:2)	PI(18:0_18:2)	C45H83O13P	862.5571	$[M+H]^+$				$[M-H]^-$	18.3	pos/neg	
PI	PI(36:3)	PI(16:0_20:3)	C45H81O13P	860.5414	$[M+Na]^+$				$[M-H]^-$	17.5	pos	neg
PI	PI(36:4)	PI(16:0_20:4)	C45H79O13P	858.5258	$[M+H]^+$				$[M-H]^-$	17.1	pos/neg	neg
PI	PI(38:3)	PI(18:0_20:3)	C47H85O13P	888.5727	$[M+H]^+$				$[M-H]^-$	18.6	pos/neg	neg
PI	PI(38:4)	PI(18:0_20:4)	C47H83O13P	886.5571	$[M+H]^+$				$[M-H]^-$	18.3	pos/neg	neg
PI	PI(38:6)	PI(16:0_22:6)	C47H79O13P	882.5258					$[M-H]^-$	16.9	pos/neg	
PI	PI(40:6)	PI(18:0_22:6)	C49H83O13P	910.5571					$[M-H]^-$	18.1	pos/neg	neg
						**Neutral Lipids**						
						**CE**						
CE	CE 18:2	CE 18:2	C45H76O2	648.5845			$[M+NH_4]^+$			26.9		
CE	CE 20:3	CE 20:3	C47H78O2	674.6002			$[M+NH_4]^+$			27.0		
CE	CE 20:4	CE 20:4	C47H76O2	672.5845			$[M+NH_4]^+$			26.2		

Lipids Subclasses	Lipids Species	Molecular Species	Molecular Formula	Monoisoto-pic Mass	$[M+H]^+$	$[M-H_2O+H]^+$	$[M+NH_4]^+$	$[M+HCOO]^-$	$[M-H]^-$	RT	Lipostar	LipidHunter
CE	CE 22:6	CE 22:6	C49H76O2	696.5845			$[M+NH_4]^+$			21.9		
**DG**												
DG	DG(34:1)	DG(16:0_18:1)	C37H70O5	594.5223			$[M+NH_4]^+$			20.8	pos	
DG	DG(34:2)	DG(16:0_18:2)	C37H68O5	592.5066			$[M+NH_4]^+$			20.1	pos	
DG	DG(36:2)	DG(18:1_18:1)	C39H72O5	620.5379			$[M+NH_4]^+$			20.9	pos	
DG	DG(36:3)	DG(18:1_18:2)	C39H70O5	618.5223			$[M+NH_4]^+$			20.2	pos	
DG	DG(36:4)	DG(18:2_18:2)	C39H68O5	616.5066			$[M+NH_4]^+$			19.4	pos	pos
DG	DG(36:4)	DG(18:1_18:3)	C39H68O5	616.5066			$[M+NH_4]^+$			19.4	pos	pos
DG	DG(38:5)	DG(18:1_20:4)	C41H70O5	642.5223			$[M+NH_4]^+$			20.0	pos	pos
DG	DG(38:6)	DG(18:2_20:4)	C41H68O5	640.5066			$[M+NH_4]^+$			19.2	pos	pos
**TG**												
TG	TG(38:0)	TG(12:0_12:0_14:0)	C41H78O6	666.5798			$[M+NH_4]^+$			22.6	pos	
TG	TG(40:0)	TG(12:0_12:0_16:0)	C43H82O6	694.6111			$[M+NH_4]^+$			23.3	pos	
TG	TG(40:0)	TG(12:0_14:0_14:0)	C43H82O6	694.6111			$[M+NH_4]^+$			23.3	pos	
TG	TG(40:0)	TG(10:0_14:0_16:0)	C43H82O6	694.6111			$[M+NH_4]^+$			23.3	pos	
TG	TG(42:0)	TG(12:0_14:0_16:0)	C45H86O6	722.6424			$[M+NH_4]^+$			23.8	pos	
TG	TG(42:0)	TG(14:0_14:0_14:0)	C45H86O6	722.6424			$[M+NH_4]^+$			23.8	pos	pos
TG	TG(42:0)	TG(12:0_12:0_18:0)	C45H86O6	722.6424			$[M+NH_4]^+$			23.8	pos	
TG	TG(42:0)	TG(10:0_16:0_16:0)	C45H86O6	722.6424			$[M+NH_4]^+$			23.8	pos	pos
TG	TG(42:1)	TG(12:0_12:0_18:1)	C45H84O6	720.6267			$[M+NH_4]^+$			23.3	pos	pos
TG	TG(42:1)	TG(12:0_14:0_16:1)	C45H84O6	720.6267			$[M+NH_4]^+$			23.3	pos	
TG	TG(42:1)	TG(12:0_14:1_16:0)	C45H84O6	720.6267			$[M+NH_4]^+$			23.3	pos	
TG	TG(42:1)	TG(14:0_14:0_14:1)	C45H84O6	720.6267			$[M+NH_4]^+$			23.3	pos	
TG	TG(43:0)	TG(12:0_15:0_16:0)	C46H88O6	736.6580			$[M+NH_4]^+$			24.1	pos	
TG	TG(43:0)	TG(14:0_14:0_15:0)	C46H88O6	736.6580			$[M+NH_4]^+$			24.1	pos	
TG	TG(43:0)	TG(11:0_16:0_16:0)	C46H88O6	736.6580			$[M+NH_4]^+$			24.1	pos	pos
TG	TG(44:0)	TG(14:0_14:0_16:0)	C47H90O6	750.6737			$[M+NH_4]^+$			24.5	pos	pos
TG	TG(44:0)	TG(12:0_16:0_16:0)	C47H90O6	750.6737			$[M+NH_4]^+$			24.5	pos	pos
TG	TG(44:0)	TG(12:0_14:0_18:0)	C47H90O6	750.6737			$[M+NH_4]^+$			24.5	pos	
TG	TG(44:1)	TG(12:0_14:0_18:1)	C47H88O6	748.6580			$[M+NH_4]^+$			23.9	pos	pos
TG	TG(44:1)	TG(12:0_16:0_16:1)	C47H88O6	748.6580			$[M+NH_4]^+$			23.9	pos	

Lipids Subclasses	Lipids Species	Molecular Species	Molecular Formula	Monoisoto-pic Mass	[M+H]$^+$	[M-H$_2$O+H]$^+$	[M+NH$_4$]$^+$	[M+HCOO]$^-$	[M-H]$^-$	RT	Lipostar	LipidHunter
TG	TG(44:1)	TG(14:0_14:0_16:1)	C47H88O6	748.6580			[M+NH$_4$]$^+$			23.9	pos	pos
TG	TG(44:1)	TG(14:0_14:1_16:0)	C47H88O6	748.6580			[M+NH$_4$]$^+$			23.9	pos	
TG	TG(44:1)	TG(12:0_14:1_18:0)	C47H88O6	748.6580			[M+NH$_4$]$^+$			23.9	pos	
TG	TG(44:2)	TG(12:0_14:1_18:1)	C47H86O6	746.6424			[M+NH$_4$]$^+$			23.4	pos	
TG	TG(44:2)	TG(12:0_14:0_18:2)	C47H86O6	746.6424			[M+NH$_4$]$^+$			23.4	pos	
TG	TG(44:2)	TG(14:0_14:1_16:1)	C47H86O6	746.6424			[M+NH$_4$]$^+$			23.4	pos	
TG	TG(44:2)	TG(12:0_16:1_16:1)	C47H86O6	746.6424			[M+NH$_4$]$^+$			23.4	pos	
TG	TG(44:2)	TG(14:1_14:1_16:0)	C47H86O6	746.6424			[M+NH$_4$]$^+$			23.4	pos	
TG	TG(45:0)	TG(14:0_15:0_16:0)	C48H92O6	764.6893			[M+NH$_4$]$^+$			24.8	pos	
TG	TG(45:0)	TG(15:0_15:0_15:0)	C48H92O6	764.6893			[M+NH$_4$]$^+$			24.8	pos	
TG	TG(45:1)	TG(12:0_15:0_18:1)	C48H90O6	762.6737			[M+NH$_4$]$^+$			24.2	pos	
TG	TG(45:1)	TG(14:0_15:0_16:1)	C48H90O6	762.6737			[M+NH$_4$]$^+$			24.2	pos	
TG	TG(45:1)	TG(12:0_16:0_17:1)	C48H90O6	762.6737			[M+NH$_4$]$^+$			24.2	pos	
TG	TG(45:1)	TG(13:0_14:0_18:1)	C48H90O6	762.6737			[M+NH$_4$]$^+$			24.2	pos	
TG	TG(45:1)	TG(13:0_16:0_16:1)	C48H90O6	762.6737			[M+NH$_4$]$^+$			24.2	pos	
TG	TG(45:1)	TG(13:0_15:0_17:1)	C48H90O6	762.6737			[M+NH$_4$]$^+$			24.2	pos	
TG	TG(45:1)	TG(14:0_14:0_17:1)	C48H90O6	762.6737			[M+NH$_4$]$^+$			24.2	pos	
TG	TG(45:2)	TG(12:0_15:0_18:2)	C48H88O6	760.6580			[M+NH$_4$]$^+$			24.8	pos	
TG	TG(46:0)	TG(14:0_16:0_16:0)	C49H94O6	778.7050			[M+NH$_4$]$^+$			25.2	pos	pos
TG	TG(46:0)	TG(15:0_15:0_16:0)	C49H94O6	778.7050			[M+NH$_4$]$^+$			25.2	pos	pos
TG	TG(46:0)	TG(14:0_14:0_18:0)	C49H94O6	778.7050			[M+NH$_4$]$^+$			25.2	pos	
TG	TG(46:1)	TG(14:0_16:0_16:1)	C49H92O6	776.6893			[M+NH$_4$]$^+$			24.5	pos	pos
TG	TG(46:1)	TG(14:0_14:0_18:1)	C49H92O6	776.6893			[M+NH$_4$]$^+$			24.5	pos	pos
TG	TG(46:1)	TG(12:0_16:0_18:1)	C49H92O6	776.6893			[M+NH$_4$]$^+$			24.5	pos	
TG	TG(46:1)	TG(14:0_14:1_18:0)	C49H92O6	776.6893			[M+NH$_4$]$^+$			24.5	pos	
TG	TG(46:1)	TG(14:1_16:0_16:0)	C49H92O6	776.6893			[M+NH$_4$]$^+$			24.5	pos	pos
TG	TG(46:1)	TG(12:0_16:1_18:0)	C49H92O6	776.6893			[M+NH$_4$]$^+$			24.5	pos	
TG	TG(46:2)	TG(12:0_16:1_18:1)	C49H90O6	774.6737			[M+NH$_4$]$^+$			24.0	pos	pos
TG	TG(46:2)	TG(12:0_16:0_18:2)	C49H90O6	774.6737			[M+NH$_4$]$^+$			24.0	pos	pos
TG	TG(46:2)	TG(14:0_14:1_18:1)	C49H90O6	774.6737			[M+NH$_4$]$^+$			24.0	pos	
TG	TG(46:2)	TG(14:1_16:0_16:1)	C49H90O6	774.6737			[M+NH$_4$]$^+$			24.0	pos	

Lipids Subclasses	Lipids Species	Molecular Species	Molecular Formula	Monoisoto-pic Mass	$[M+H]^+$	$[M-H_2O+H]^+$	$[M+NH_4]^+$	$[M+HCOO]^-$	$[M-H]^-$	RT	Lipostar	LipidHunter
TG	TG(46:2)	TG(14:0_14:0_18:2)	C49H90O6	774.6737			$[M+NH_4]^+$			24.0	pos	
TG	TG(46:2)	TG(14:0_16:1_16:1)	C49H90O6	774.6737			$[M+NH_4]^+$			24.0	pos	
TG	TG(46:2)	TG(10:0_18:1_18:1)	C49H90O6	774.6737			$[M+NH_4]^+$			24.0	pos	pos
TG	TG(46:3)	TG(12:0_16:1_18:2)	C49H88O6	772.6580			$[M+NH_4]^+$			23.5	pos	
TG	TG(46:3)	TG(14:0_14:1_18:2)	C49H88O6	772.6580			$[M+NH_4]^+$			23.5	pos	
TG	TG(46:3)	TG(14:1_14:1_18:1)	C49H88O6	772.6580			$[M+NH_4]^+$			23.5	pos	
TG	TG(46:3)	TG(14:1_16:1_16:1)	C49H88O6	772.6580			$[M+NH_4]^+$			23.5	pos	
TG	TG(46:3)	TG(10:0_18:1_18:2)	C49H88O6	772.6580			$[M+NH_4]^+$			23.5	pos	pos
TG	TG(46:4)	TG(14:1_14:1_18:2)	C49H86O6	770.6424			$[M+NH_4]^+$			23.0	pos	
TG	TG(47:0)	TG(15:0_16:0_16:0)	C50H96O6	792.7206			$[M+NH_4]^+$			25.7	pos	
TG	TG(47:0)	TG(14:0_16:0_17:0)	C50H96O6	792.7206			$[M+NH_4]^+$			25.7	pos	
TG	TG(47:0)	TG(14:0_15:0_18:0)	C50H96O6	792.7206			$[M+NH_4]^+$			25.7	pos	
TG	TG(47:0)	TG(15:0_15:0_17:0)	C50H96O6	792.7206			$[M+NH_4]^+$			25.7	pos	pos
TG	TG(47:1)	TG(15:0_16:0_16:1)	C50H94O6	790.7050			$[M+NH_4]^+$			24.9	pos	pos
TG	TG(47:1)	TG(15:0_15:0_17:1)	C50H94O6	790.7050			$[M+NH_4]^+$			24.9	pos	
TG	TG(47:1)	TG(15:0_15:1_17:0)	C50H94O6	790.7050			$[M+NH_4]^+$			24.9	pos	
TG	TG(47:1)	TG(14:1_16:0_17:0)	C50H94O6	790.7050			$[M+NH_4]^+$			24.9	pos	
TG	TG(47:1)	TG(15:1_16:0_16:0)	C50H94O6	790.7050			$[M+NH_4]^+$			24.9	pos	
TG	TG(47:1)	TG(14:0_15:0_18:1)	C50H94O6	790.7050			$[M+NH_4]^+$			24.9	pos	pos
TG	TG(47:2)	TG(14:0_15:0_18:2)	C50H92O6	788.6893			$[M+NH_4]^+$			24.3	pos	
TG	TG(47:2)	TG(13:0_16:0_18:2)	C50H92O6	788.6893			$[M+NH_4]^+$			24.3	pos	
TG	TG(47:2)	TG(15:0_16:1_16:1)	C50H92O6	788.6893			$[M+NH_4]^+$			24.3	pos	
TG	TG(47:2)	TG(14:1_15:0_18:1)	C50H92O6	788.6893			$[M+NH_4]^+$			24.3	pos	
TG	TG(47:2)	TG(13:0_16:1_18:1)	C50H92O6	788.6893			$[M+NH_4]^+$			24.3	pos	
TG	TG(48:0)	TG(14:0_16:0_18:0)	C51H98O6	806.7363			$[M+NH_4]^+$			26.2	pos	pos
TG	TG(48:0)	TG(16:0_16:0_16:0)	C51H98O6	806.7363			$[M+NH_4]^+$			26.2	pos	pos
TG	TG(48:1)	TG(14:0_16:0_18:1)	C51H96O6	804.7206			$[M+NH_4]^+$			25.3	pos	pos
TG	TG(48:1)	TG(16:0_16:0_16:1)	C51H96O6	804.7206			$[M+NH_4]^+$			25.3	pos	pos
TG	TG(48:1)	TG(14:0_16:1_18:0)	C51H96O6	804.7206			$[M+NH_4]^+$			25.3	pos	
TG	TG(48:2)	TG(14:0_16:1_18:1)	C51H94O6	802.7050			$[M+NH_4]^+$			24.5	pos	pos
TG	TG(48:2)	TG(12:0_18:1_18:1)	C51H94O6	802.7050			$[M+NH_4]^+$			24.5	pos	pos

Lipids Subclasses	Lipids Species	Molecular Species	Molecular Formula	Monoisoto -pic Mass	$[M+H]^+$	$[M-H_2O+H]^+$	$[M+NH_4]^+$	$[M+HCOO]^-$	$[M-H]^-$	RT	Lipostar	LipidHunter
TG	TG(48:2)	TG(14:1_16:0_18:1)	C51H94O6	802.7050			$[M+NH_4]^+$			24.5	pos	
TG	TG(48:2)	TG(16:0_16:1_16:1)	C51H94O6	802.7050			$[M+NH_4]^+$			24.5	pos	pos
TG	TG(48:2)	TG(14:0_16:0_18:2)	C51H94O6	802.7050			$[M+NH_4]^+$			24.5	pos	pos
TG	TG(48:3)	TG(12:0_18:1_18:2)	C51H92O6	800.6893			$[M+NH_4]^+$			24.0	pos	pos
TG	TG(48:3)	TG(14:0_16:1_18:2)	C51H92O6	800.6893			$[M+NH_4]^+$			24.0	pos	
TG	TG(48:3)	TG(14:1_16:0_18:2)	C51H92O6	800.6893			$[M+NH_4]^+$			24.0	pos	
TG	TG(48:3)	TG(14:1_16:1_18:1)	C51H92O6	800.6893			$[M+NH_4]^+$			24.0	pos	
TG	TG(48:3)	TG(16:1_16:1_16:1)	C51H92O6	800.6893			$[M+NH_4]^+$			24.0	pos	pos
TG	TG(49:0)	TG(16:0_16:0_17:0)	C52H100O6	820.7519			$[M+NH_4]^+$			26.5	pos	pos
TG	TG(49:0)	TG(15:0_16:0_18:0)	C52H100O6	820.7519			$[M+NH_4]^+$			26.5	pos	pos
TG	TG(49:0)	TG(15:0_17:0_17:0)	C52H100O6	820.7519			$[M+NH_4]^+$			26.5	pos	
TG	TG(49:0)	TG(14:0_17:0_18:0)	C52H100O6	820.7519			$[M+NH_4]^+$			26.5	pos	
TG	TG(49:1)	TG(15:0_16:0_18:1)	C52H98O6	818.7363			$[M+NH_4]^+$			25.7	pos	pos
TG	TG(49:1)	TG(16:0_16:0_17:1)	C52H98O6	818.7363			$[M+NH_4]^+$			25.7	pos	pos
TG	TG(49:1)	TG(16:0_16:1_17:0)	C52H98O6	818.7363			$[M+NH_4]^+$			25.7	pos	
TG	TG(49:1)	TG(14:0_17:0_18:1)	C52H98O6	818.7363			$[M+NH_4]^+$			25.7	pos	
TG	TG(49:1)	TG(15:0_17:0_17:1)	C52H98O6	818.7363			$[M+NH_4]^+$			25.7	pos	
TG	TG(49:1)	TG(15:0_16:1_18:0)	C52H98O6	818.7363			$[M+NH_4]^+$			25.7	pos	
TG	TG(49:1)	TG(14:0_17:1_18:0)	C52H98O6	818.7363			$[M+NH_4]^+$			25.7	pos	
TG	TG(49:2)	TG(15:0_16:1_18:1)	C52H96O6	816.7206			$[M+NH_4]^+$			24.9	pos	
TG	TG(49:2)	TG(16:0_16:1_17:1)	C52H96O6	816.7206			$[M+NH_4]^+$			24.9	pos	
TG	TG(49:2)	TG(15:0_16:0_18:2)	C52H96O6	816.7206			$[M+NH_4]^+$			24.9	pos	pos
TG	TG(49:2)	TG(14:0_17:1_18:1)	C52H96O6	816.7206			$[M+NH_4]^+$			24.9	pos	
TG	TG(49:2)	TG(15:1_16:0_18:1)	C52H96O6	816.7206			$[M+NH_4]^+$			24.9	pos	
TG	TG(49:2)	TG(15:0_17:1_17:1)	C52H96O6	816.7206			$[M+NH_4]^+$			24.9	pos	
TG	TG(49:2)	TG(13:0_18:1_18:1)	C52H96O6	816.7206			$[M+NH_4]^+$			24.9	pos	
TG	TG(49:2)	TG(16:1_16:1_17:0)	C52H96O6	816.7206			$[M+NH_4]^+$			24.9	pos	
TG	TG(49:2)	TG(15:1_17:0_17:1)	C52H96O6	816.7206			$[M+NH_4]^+$			24.9	pos	
TG	TG(49:3)	TG(15:0_16:1_18:2)	C52H94O6	814.7050			$[M+NH_4]^+$			24.3	pos	pos
TG	TG(49:3)	TG(15:1_16:1_18:1)	C52H94O6	814.7050			$[M+NH_4]^+$			24.3	pos	
TG	TG(49:3)	TG(15:1_16:0_18:2)	C52H94O6	814.7050			$[M+NH_4]^+$			24.3	pos	

Lipids Subclasses	Lipids Species	Molecular Species	Molecular Formula	Monoisoto-pic Mass	$[M+H]^+$	$[M-H_2O+H]^+$	$[M+NH_4]^+$	$[M+HCOO]^-$	$[M-H]^-$	RT	Lipostar	LipidHunter
TG	TG(49:3)	TG(14:0_17:1_18:2)	C52H94O6	814.7050			$[M+NH_4]^+$			24.3	pos	
TG	TG(49:3)	TG(16:1_16:1_17:1)	C52H94O6	814.7050			$[M+NH_4]^+$			24.3	pos	
TG	TG(49:3)	TG(15:0_16:0_18:3)	C52H94O6	814.7050			$[M+NH_4]^+$			24.3	pos	
TG	TG(49:3)	TG(15:1_17:1_17:1)	C52H94O6	814.7050			$[M+NH_4]^+$			24.3	pos	
TG	TG(50:0)	TG(16:0_16:0_18:0)	C53H102O6	834.7676			$[M+NH_4]^+$			27.3	pos	pos
TG	TG(50:0)	TG(14:0_18:0_18:0)	C53H102O6	834.7676			$[M+NH_4]^+$			27.3	pos	pos
TG	TG(50:1)	TG(16:0_16:0_18:1)	C53H100O6	832.7519			$[M+NH_4]^+$			26.2	pos	pos
TG	TG(50:1)	TG(16:0_16:1_18:0)	C53H100O6	832.7519			$[M+NH_4]^+$			26.2	pos	
TG	TG(50:2)	TG(16:0_16:1_18:1)	C53H98O6	830.7363			$[M+NH_4]^+$			25.3	pos	pos
TG	TG(50:2)	TG(14:0_18:1_18:1)	C53H98O6	830.7363			$[M+NH_4]^+$			25.3	pos	pos
TG	TG(50:2)	TG(16:0_16:0_18:2)	C53H98O6	830.7363			$[M+NH_4]^+$			25.3	pos	pos
TG	TG(50:3)	TG(16:0_16:1_18:2)	C53H96O6	828.7206			$[M+NH_4]^+$			24.7	pos	pos
TG	TG(50:3)	TG(14:0_18:1_18:2)	C53H96O6	828.7206			$[M+NH_4]^+$			24.7	pos	pos
TG	TG(50:3)	TG(16:1_16:1_18:1)	C53H96O6	828.7206			$[M+NH_4]^+$			24.7	pos	pos
TG	TG(50:3)	TG(14:1_18:1_18:1)	C53H96O6	828.7206			$[M+NH_4]^+$			24.7	pos	pos
TG	TG(50:4)	TG(14:0_18:2_18:2)	C53H94O6	826.7050			$[M+NH_4]^+$			24.1	pos	pos
TG	TG(50:4)	TG(16:1_16:1_18:2)	C53H94O6	826.7050			$[M+NH_4]^+$			24.1	pos	pos
TG	TG(50:4)	TG(14:1_18:1_18:2)	C53H94O6	826.7050			$[M+NH_4]^+$			24.1	pos	
TG	TG(50:4)	TG(14:0_16:0_20:4)	C53H94O6	826.7050			$[M+NH_4]^+$			24.1	pos	pos
TG	TG(50:4)	TG(16:0_16:0_18:4)	C53H94O6	826.7050			$[M+NH_4]^+$			24.1	pos	pos
TG	TG(50:4)	TG(16:0_16:1_18:3)	C53H94O6	826.7050			$[M+NH_4]^+$			24.1	pos	pos
TG	TG(50:5)	TG(14:0_18:2_18:3)	C53H92O6	824.6893			$[M+NH_4]^+$			23.8	pos	pos
TG	TG(50:5)	TG(14:1_18:1_18:3)	C53H92O6	824.6893			$[M+NH_4]^+$			23.8	pos	
TG	TG(50:5)	TG(16:1_16:1_18:3)	C53H92O6	824.6893			$[M+NH_4]^+$			23.8	pos	
TG	TG(50:5)	TG(14:1_18:2_18:2)	C53H92O6	824.6893			$[M+NH_4]^+$			23.8	pos	
TG	TG(50:6)	TG(12:0_18:1_20:5)	C53H90O6	822.6737			$[M+NH_4]^+$			23.4	pos	
TG	TG(51:1)	TG(16:0_17:0_18:1)	C54H102O6	846.7676			$[M+NH_4]^+$			26.7	pos	pos
TG	TG(51:1)	TG(15:0_18:0_18:1)	C54H102O6	846.7676			$[M+NH_4]^+$			26.7	pos	
TG	TG(51:1)	TG(16:0_17:1_18:0)	C54H102O6	846.7676			$[M+NH_4]^+$			26.7	pos	
TG	TG(51:1)	TG(16:0_16:0_19:1)	C54H102O6	846.7676			$[M+NH_4]^+$			26.7	pos	pos
TG	TG(51:1)	TG(15:0_16:0_20:1)	C54H102O6	846.7676			$[M+NH_4]^+$			26.7	pos	

Lipids Subclasses	Lipids Species	Molecular Species	Molecular Formula	Monoisoto-pic Mass	$[M+H]^+$	$[M-H_2O+H]^+$	$[M+NH_4]^+$	$[M+HCOO]^-$	$[M-H]^-$	RT	Lipostar	LipidHunter
TG	TG(51:1)	TG(15:0_17:0_19:1)	C54H102O6	846.7676			$[M+NH_4]^+$			26.7	pos	
TG	TG(51:1)	TG(17:0_17:0_17:1)	C54H102O6	846.7676			$[M+NH_4]^+$			26.7	pos	
TG	TG(51:2)	TG(15:0_18:1_18:1)	C54H102O6	844.7519			$[M+NH_4]^+$			25.7	pos	pos
TG	TG(51:2)	TG(16:0_17:1_18:1)	C54H102O6	844.7519			$[M+NH_4]^+$			25.7	pos	pos
TG	TG(51:3)	TG(15:0_18:1_18:2)	C54H98O6	842.7363			$[M+NH_4]^+$			25.1	pos	pos
TG	TG(51:3)	TG(16:0_17:1_18:2)	C54H98O6	842.7363			$[M+NH_4]^+$			25.1	pos	
TG	TG(51:3)	TG(16:1_17:1_18:1)	C54H98O6	842.7363			$[M+NH_4]^+$			25.1	pos	
TG	TG(51:3)	TG(16:0_17:2_18:1)	C54H98O6	842.7363			$[M+NH_4]^+$			25.1	pos	
TG	TG(51:3)	TG(16:1_17:0_18:2)	C54H98O6	842.7363			$[M+NH_4]^+$			25.1	pos	
TG	TG(51:3)	TG(15:1_18:1_18:1)	C54H98O6	842.7363			$[M+NH_4]^+$			25.1	pos	
TG	TG(51:3)	TG(17:0_17:1_17:2)	C54H98O6	842.7363			$[M+NH_4]^+$			25.1	pos	
TG	TG(51:3)	TG(17:1_17:1_17:1)	C54H98O6	842.7363			$[M+NH_4]^+$			25.1	pos	
TG	TG(51:4)	TG(15:0_18:2_18:2)	C54H96O6	840.7206			$[M+NH_4]^+$			24.5	pos	pos
TG	TG(51:4)	TG(16:1_17:1_18:2)	C54H96O6	840.7206			$[M+NH_4]^+$			24.5	pos	
TG	TG(51:4)	TG(15:1_18:1_18:2)	C54H96O6	840.7206			$[M+NH_4]^+$			24.5	pos	
TG	TG(51:4)	TG(16:0_17:2_18:2)	C54H96O6	840.7206			$[M+NH_4]^+$			24.5	pos	
TG	TG(51:4)	TG(15:0_18:1_18:3)	C54H96O6	840.7206			$[M+NH_4]^+$			24.5	pos	
TG	TG(52:1)	TG(16:0_18:0_18:1)	C55H104O6	860.7832			$[M+NH_4]^+$			27.3	pos	pos
TG	TG(52:1)	TG(16:0_16:0_20:1)	C55H104O6	860.7832			$[M+NH_4]^+$			27.3	pos	pos
TG	TG(52:1)	TG(16:1_18:0_18:0)	C55H104O6	860.7832			$[M+NH_4]^+$			27.3	pos	pos
TG	TG(52:2)	TG(16:0_18:1_18:1)	C55H102O6	858.7676			$[M+NH_4]^+$			26.2	pos	pos
TG	TG(52:3)	TG(16:0_18:1_18:2)	C55H100O6	856.7519			$[M+NH_4]^+$			25.5	pos	pos
TG	TG(52:3)	TG(16:1_18:1_18:1)	C55H100O6	856.7519			$[M+NH_4]^+$			25.5	pos	pos
TG	TG(52:4)	TG(16:0_18:2_18:2)	C55H98O6	854.7363			$[M+NH_4]^+$			24.9	pos	pos
TG	TG(52:4)	TG(16:1_18:1_18:2)	C55H98O6	854.7363			$[M+NH_4]^+$			24.9	pos	
TG	TG(52:4)	TG(16:0_18:1_18:3)	C55H98O6	854.7363			$[M+NH_4]^+$			24.9	pos	pos
TG	TG(52:4)	TG(16:0_16:1_20:3)	C55H98O6	854.7363			$[M+NH_4]^+$			24.9	pos	
TG	TG(52:4)	TG(16:0_16:0_20:4)	C55H98O6	854.7363			$[M+NH_4]^+$			24.9	pos	pos
TG	TG(52:5)	TG(16:0_18:2_18:3)	C55H96O6	852.7206			$[M+NH_4]^+$			24.4	pos	pos
TG	TG(52:5)	TG(16:1_18:1_18:3)	C55H96O6	852.7206			$[M+NH_4]^+$			24.4	pos	
TG	TG(52:5)	TG(16:0_16:1_20:4)	C55H96O6	852.7206			$[M+NH_4]^+$			24.4	pos	

Lipids Subclasses	Lipids Species	Molecular Species	Molecular Formula	Monoisoto-pic Mass	$[M+H]^+$	$[M-H_2O+H]^+$	$[M+NH_4]^+$	$[M+HCOO]^-$	$[M-H]^-$	RT	Lipostar	LipidHunter
TG	TG(52:5)	TG(16:0_18:1_18:4)	C55H96O6	852.7206			$[M+NH_4]^+$			24.4	pos	
TG	TG(52:5)	TG(16:1_18:2_18:2)	C55H96O6	852.7206			$[M+NH_4]^+$			24.4	pos	pos
TG	TG(52:6)	TG(16:1_16:1_20:4)	C55H94O6	850.7050			$[M+NH_4]^+$			23.8	pos	pos
TG	TG(52:6)	TG(16:1_18:2_18:3)	C55H94O6	850.7050			$[M+NH_4]^+$			23.8	pos	pos
TG	TG(53:2)	TG(17:0_18:1_18:1)	C56H104O6	872.7832			$[M+NH_4]^+$			26.8	pos	pos
TG	TG(53:2)	TG(16:0_18:1_19:1)	C56H104O6	872.7832			$[M+NH_4]^+$			26.8	pos	pos
TG	TG(53:2)	TG(17:1_18:0_18:1)	C56H104O6	872.7832			$[M+NH_4]^+$			26.8	pos	
TG	TG(53:2)	TG(17:0_17:1_19:1)	C56H104O6	872.7832			$[M+NH_4]^+$			26.8	pos	
TG	TG(53:2)	TG(17:0_18:0_18:2)	C56H104O6	872.7832			$[M+NH_4]^+$			26.8	pos	
TG	TG(53:3)	TG(17:0_18:1_18:2)	C56H102O6	870.7676			$[M+NH_4]^+$			25.8	pos	pos
TG	TG(53:3)	TG(17:1_18:1_18:1)	C56H102O6	870.7676			$[M+NH_4]^+$			25.8	pos	pos
TG	TG(53:3)	TG(17:2_18:0_18:1)	C56H102O6	870.7676			$[M+NH_4]^+$			25.8	pos	
TG	TG(53:3)	TG(17:1_18:0_18:2)	C56H102O6	870.7676			$[M+NH_4]^+$			25.8	pos	
TG	TG(53:4)	TG(17:0_18:2_18:2)	C56H100O6	868.7519			$[M+NH_4]^+$			25.3	pos	pos
TG	TG(53:4)	TG(17:1_18:1_18:2)	C56H100O6	868.7519			$[M+NH_4]^+$			25.3	pos	pos
TG	TG(53:4)	TG(17:0_18:1_18:3)	C56H100O6	868.7519			$[M+NH_4]^+$			25.3	pos	
TG	TG(53:4)	TG(17:2_18:0_18:2)	C56H100O6	868.7519			$[M+NH_4]^+$			25.3	pos	
TG	TG(53:4)	TG(17:2_18:1_18:1)	C56H100O6	868.7519			$[M+NH_4]^+$			25.3	pos	
TG	TG(53:5)	TG(17:1_18:2_18:2)	C56H98O6	866.7363			$[M+NH_4]^+$			24.5	pos	
TG	TG(53:5)	TG(17:0_18:2_18:3)	C56H98O6	866.7363			$[M+NH_4]^+$			24.5	pos	
TG	TG(53:5)	TG(17:1_18:1_18:3)	C56H98O6	866.7363			$[M+NH_4]^+$			24.5	pos	
TG	TG(53:5)	TG(15:0_18:1_20:4)	C56H98O6	866.7363			$[M+NH_4]^+$			24.9	pos	
TG	TG(54:1)	TG(18:0_18:0_18:1)	C57H108O6	888.8145			$[M+NH_4]^+$			23.3	pos	
TG	TG(54:1)	TG(16:0_18:0_20:1)	C57H108O6	888.8145			$[M+NH_4]^+$			23.3	pos	
TG	TG(54:1)	TG(16:0_18:1_20:0)	C57H108O6	888.8145			$[M+NH_4]^+$			23.3	pos	
TG	TG(54:2)	TG(18:0_18:1_18:1)	C57H106O6	886.7989			$[M+NH_4]^+$			27.4	pos	pos
TG	TG(54:2)	TG(16:0_18:1_20:1)	C57H106O6	886.7989			$[M+NH_4]^+$			27.4	pos	pos
TG	TG(54:3)	TG(16:0_18:1_20:2)	C57H104O6	884.7832			$[M+NH_4]^+$			26.3	pos	pos
TG	TG(54:3)	TG(18:0_18:1_18:2)	C57H104O6	884.7832			$[M+NH_4]^+$			26.3	pos	pos
TG	TG(54:3)	TG(18:1_18:1_18:1)	C57H104O6	884.7832			$[M+NH_4]^+$			26.3	pos	pos
TG	TG(54:4)	TG(18:1_18:1_18:2)	C57H102O6	882.7676			$[M+NH_4]^+$			25.5	pos	pos

Lipids Subclasses	Lipids Species	Molecular Species	Molecular Formula	Monoisoto -pic Mass	$[M+H]^+$	$[M-H_2O+H]^+$	$[M+NH_4]^+$	$[M+HCOO]^-$	$[M-H]^-$	RT	Lipostar	LipidHunter
TG	TG(54:4)	TG(16:0_18:2_20:2)	C57H102O6	882.7676			$[M+NH_4]^+$			25.5	pos	
TG	TG(54:5)	TG(18:1_18:2_18:2)	C57H100O6	880.7519			$[M+NH_4]^+$			24.9	pos	pos
TG	TG(54:5)	TG(18:0_18:2_18:3)	C57H100O6	880.7519			$[M+NH_4]^+$			24.9	pos	
TG	TG(54:5)	TG(18:1_18:1_18:3)	C57H100O6	880.7519			$[M+NH_4]^+$			24.9	pos	pos
TG	TG(54:5)	TG(16:0_18:1_20:4)	C57H100O6	880.7519			$[M+NH_4]^+$			24.9	pos	pos
TG	TG(54:6)	TG(18:1_18:2_18:3)	C57H98O6	878.7363			$[M+NH_4]^+$			24.3	pos	
TG	TG(54:6)	TG(18:2_18:2_18:2)	C57H98O6	878.7363			$[M+NH_4]^+$			24.3	pos	pos
TG	TG(54:6)	TG(18:0_18:3_18:3)	C57H98O6	878.7363			$[M+NH_4]^+$			24.3	pos	
TG	TG(54:6)	TG(18:1_18:1_18:4)	C57H98O6	878.7363			$[M+NH_4]^+$			24.3	pos	
TG	TG(54:6)	TG(18:0_18:2_18:4)	C57H98O6	878.7363			$[M+NH_4]^+$			24.3	pos	
TG	TG(54:6)	TG(16:0_18:2_20:4)	C57H98O6	878.7363			$[M+NH_4]^+$			24.3	pos	pos
TG	TG(54:7)	TG(18:1_18:3_18:3)	C57H96O6	876.7206			$[M+NH_4]^+$			23.9		pos
TG	TG(54:7)	TG(18:2_18:2_18:3)	C57H96O6	876.7206			$[M+NH_4]^+$			23.9		pos
TG	TG(55:3)	TG(18:1_18:1_19:1)	C58H106O6	898.7989			$[M+NH_4]^+$			26.8	pos	pos
TG	TG(55:4)	TG(18:1_18:2_19:1)	C58H104O6	896.7832			$[M+NH_4]^+$			26.0	pos	pos
TG	TG(55:5)	TG(18:2_18:2_19:1)	C58H102O6	894.7676			$[M+NH_4]^+$			25.3	pos	pos
TG	TG(55:6)	TG(17:1_18:1_20:4)	C58H100O6	892.7519			$[M+NH_4]^+$			24.9	pos	
TG	TG(55:6)	TG(15:0_18:1_22:5)	C58H100O6	892.7519			$[M+NH_4]^+$			24.9	pos	
TG	TG(55:7)	TG (15:0_18:1_22:6)	C58H98O6	890.7363			$[M+NH_4]^+$			24.7	pos	
TG	TG(56:2)	TG(16:0_18:1_22:1)	C59H110O6	914.8302			$[M+NH_4]^+$			23.2	pos	
TG	TG(56:2)	TG(18:1_18:1_20:0)	C59H110O6	914.8302			$[M+NH_4]^+$			23.2	pos	
TG	TG(56:2)	TG(16:0_20:1_20:1)	C59H110O6	914.8302			$[M+NH_4]^+$			23.2	pos	
TG	TG(56:2)	TG(18:0_18:2_20:0)	C59H110O6	914.8302			$[M+NH_4]^+$			23.2	pos	
TG	TG(56:3)	TG(18:1_18:1_20:1)	C59H108O6	912.8145			$[M+NH_4]^+$			27.3	pos	pos
TG	TG(56:4)	TG(18:0_18:1_20:3)	C59H106O6	910.7989			$[M+NH_4]^+$			26.7	pos	pos
TG	TG(56:4)	TG(16:0_18:1_22:3)	C59H106O6	910.7989			$[M+NH_4]^+$			26.7	pos	
TG	TG(56:4)	TG(18:1_18:1_20:2)	C59H106O6	910.7989			$[M+NH_4]^+$			26.4	pos	pos
TG	TG(56:4)	TG(18:0_18:2_20:2)	C59H106O6	910.7989			$[M+NH_4]^+$			26.7	pos	
TG	TG(56:4)	TG(18:1_18:2_20:1)	C59H106O6	910.7989			$[M+NH_4]^+$			26.4	pos	pos
TG	TG(56:5)	TG(16:0_18:1_22:4)	C59H104O6	908.7832			$[M+NH_4]^+$			25.9	pos	pos
TG	TG(56:5)	TG(18:1_18:1_20:3)	C59H104O6	908.7832			$[M+NH_4]^+$			25.9	pos	pos

Lipids Subclasses	Lipids Species	Molecular Species	Molecular Formula	Monoisoto-pic Mass	$[M+H]^+$	$[M-H_2O+H]^+$	$[M+NH_4]^+$	$[M+HCOO]^-$	$[M-H]^-$	RT	Lipostar	LipidHunter
TG	TG(56:5)	TG(16:0_20:2_20:3)	C59H104O6	908.7839			$[M+NH_4]^+$			25.9	pos	
TG	TG(56:5)	TG(18:0_18:1_20:4)	C59H104O6	908.7832			$[M+NH_4]^+$			25.9	pos	pos
TG	TG(56:6)	TG(16:0_18:1_22:5)	C59H102O6	906.7676			$[M+NH_4]^+$			25.3	pos	pos
TG	TG(56:6)	TG(18:1_18:1_20:4)	C59H102O6	906.7676			$[M+NH_4]^+$			25.3	pos	pos
TG	TG(56:6)	TG(16:0_18:2_22:4)	C59H102O6	906.7676			$[M+NH_4]^+$			25.3	pos	pos
TG	TG(56:6)	TG(16:0_20:3_20:3)	C59H102O6	906.7676			$[M+NH_4]^+$			25.3	pos	
TG	TG(56:6)	TG(18:0_18:2_20:4)	C59H102O6	906.7676			$[M+NH_4]^+$			25.3	pos	pos
TG	TG(56:7)	TG(18:1_18:2_20:4)	C59H100O6	904.7519			$[M+NH_4]^+$			24.7	pos	pos
TG	TG(56:7)	TG(16:0_18:2_22:5)	C59H100O6	904.7519			$[M+NH_4]^+$			24.7	pos	
TG	TG(56:7)	TG(18:1_18:1_20:5)	C59H100O6	904.7519			$[M+NH_4]^+$			24.7	pos	pos
TG	TG(56:7)	TG(16:1_18:1_22:5)	C59H100O6	904.7519			$[M+NH_4]^+$			24.7	pos	
TG	TG(56:7)	TG(16:0_18:1_22:6)	C59H100O6	904.7519			$[M+NH_4]^+$			24.7	pos	pos
TG	TG(56:8)	TG(16:0_18:2_22:6)	C59H98O6	902.7363			$[M+NH_4]^+$			24.5	pos	pos
TG	TG(56:8)	TG(16:0_20:4_20:4)	C59H98O6	902.7363			$[M+NH_4]^+$			24.5	pos	
TG	TG(56:8)	TG(18:2_18:2_20:4)	C59H98O6	902.7363			$[M+NH_4]^+$			24.5	pos	pos
TG	TG(58:10)	TG(18:2_20:4_20:4)	C61H98O6	926.7363			$[M+NH_4]^+$			24.0	pos	
TG	TG(58:10)	TG(18:2_18:2_22:6)	C61H98O6	926.7363			$[M+NH_4]^+$			24.0	pos	
TG	TG(58:5)	TG(18:1_18:1_22:3)	C61H108O6	936.8145			$[M+NH_4]^+$			26.8	pos	
TG	TG(58:6)	TG(18:1_18:1_22:4)	C61H106O6	934.7989			$[M+NH_4]^+$			26.0	pos	
TG	TG(58:7)	TG(18:1_18:1_22:5)	C61H104O6	932.7832			$[M+NH_4]^+$			25.6	pos	pos
TG	TG(58:7)	TG(18:0_18:2_22:5)	C61H104O6	932.7832			$[M+NH_4]^+$			25.6	pos	
TG	TG(58:7)	TG(18:1_18:2_22:4)	C61H104O6	932.7832			$[M+NH_4]^+$			25.6	pos	
TG	TG(58:7)	TG(18:1_20:3_20:3)	C61H104O6	932.7832			$[M+NH_4]^+$			25.6	pos	
TG	TG(58:8)	TG(18:1_18:2_22:5)	C61H102O6	930.7676			$[M+NH_4]^+$			24.8	pos	
TG	TG(58:8)	TG(18:2_18:2_22:4)	C61H102O6	930.7676			$[M+NH_4]^+$			24.8	pos	
TG	TG(58:8)	TG(18:1_18:1_22:6)	C61H102O6	930.7676			$[M+NH_4]^+$			24.8	pos	pos
TG	TG(58:9)	TG(18:1_18:2_22:6)	C61H100O6	928.7519			$[M+NH_4]^+$			24.5	pos	pos
TG	TG(58:9)	TG(18:1_20:4_20:4)	C61H100O6	928.7519			$[M+NH_4]^+$			24.5	pos	pos
TG	TG(58:9)	TG(18:2_20:3_20:4)	C61H100O6	928.7510			$[M+NH_4]^+$			24.5	pos	
TG	TG(58:9)	TG(18:2_18:2_22:5)	C61H100O6	928.7519			$[M+NH_4]^+$			24.5	pos	
TG	TG(60:10)	TG(18:1_20:4_22:5)	C63H102O6	954.7676			$[M+NH_4]^+$			24.8	pos	

Lipids Subclasses	Lipids Species	Molecular Species	Molecular Formula	Monoisoto-pic Mass	$[M+H]^+$	$[M-H_2O+H]^+$	$[M+NH_4]^+$	$[M+HCOO]^-$	$[M-H]^-$	RT	Lipostar	LipidHunter
TG	TG(60:10)	TG(18:0_20:4_22:6)	C63H102O6	954.7676			$[M+NH_4]^+$			24.8	pos	
TG	TG(60:10)	TG(16:1_22:4_22:5)	C63H102O6	954.7676			$[M+NH_4]^+$			24.8	pos	
TG	TG(60:10)	TG(18:4_20:1_22:5)	C63H102O6	954.7676			$[M+NH_4]^+$			24.8	pos	
TG	TG(60:10)	TG(20:2_20:4_20:4)	C63H102O6	954.7676			$[M+NH_4]^+$			24.8	pos	
TG	TG(60:10)	TG(16:0_22:4_22:6)	C63H102O6	954.7676			$[M+NH_4]^+$			24.8	pos	
TG	TG(60:11)	TG(18:3_20:3_22:5)	C63H100O6	952.7519			$[M+NH_4]^+$			24.3	pos	
TG	TG(60:12)	TG(20:4_20:4_20:4)	C63H98O6	950.7363			$[M+NH_4]^+$			23.8	pos	
TG	TG(60:3)	TG(18:1_20:1_22:1)	C63H116O6	968.8771			$[M+NH_4]^+$			25.0	pos	
TG	TG(60:3)	TG(20:1_20:1_20:1)	C63H116O6	968.8771			$[M+NH_4]^+$			25.0	pos	
TG	TG(62:13)	TG(18:1_22:6_22:6)	C65H100O6	976.7519			$[M+NH_4]^+$			24.2	pos	
TG	TG(62:14)	TG(20:4_20:4_22:6)	C65H98O6	974.7363			$[M+NH_4]^+$			23.7	pos	
